# Ferroptosis in cancer: from molecular mechanisms to therapeutic strategies

**DOI:** 10.1038/s41392-024-01769-5

**Published:** 2024-03-08

**Authors:** Qian Zhou, Yu Meng, Daishi Li, Lei Yao, Jiayuan Le, Yihuang Liu, Yuming Sun, Furong Zeng, Xiang Chen, Guangtong Deng

**Affiliations:** 1 Department of Dermatology, Xiangya Hospital, Central South University, 87 Xiangya Road, Changsha, 410008 Hunan Province China; 2 National Engineering Research Center of Personalized Diagnostic and Therapeutic Technology, 87 Xiangya Road, Changsha, 410008 Hunan Province China; 3 Furong Laboratory, 87 Xiangya Road, Changsha, 410008 Hunan Province China; 4 Hunan Key Laboratory of Skin Cancer and Psoriasis, Hunan Engineering Research Center of Skin Health and Disease, Xiangya Hospital, Central South University, 87 Xiangya Road, Changsha, 410008 Hunan Province China; 5 National Clinical Research Center for Geriatric Disorders, Xiangya Hospital, 87 Xiangya Road, Changsha, 410008 Hunan Province China; 6 Department of General Surgery, Xiangya Hospital, Central South University, 87 Xiangya Road, Changsha, 410008 Hunan Province China; 7 Department of Plastic and Cosmetic Surgery, Xiangya Hospital, Central South University, 87 Xiangya Road, Changsha, 410008 Hunan Province China; 8 Department of Oncology, Xiangya Hospital, Central South University, 87 Xiangya Road, Changsha, 410008 Hunan Province China

**Keywords:** Cancer, Cell biology

## Abstract

Ferroptosis is a non-apoptotic form of regulated cell death characterized by the lethal accumulation of iron-dependent membrane-localized lipid peroxides. It acts as an innate tumor suppressor mechanism and participates in the biological processes of tumors. Intriguingly, mesenchymal and dedifferentiated cancer cells, which are usually resistant to apoptosis and traditional therapies, are exquisitely vulnerable to ferroptosis, further underscoring its potential as a treatment approach for cancers, especially for refractory cancers. However, the impact of ferroptosis on cancer extends beyond its direct cytotoxic effect on tumor cells. Ferroptosis induction not only inhibits cancer but also promotes cancer development due to its potential negative impact on anticancer immunity. Thus, a comprehensive understanding of the role of ferroptosis in cancer is crucial for the successful translation of ferroptosis therapy from the laboratory to clinical applications. In this review, we provide an overview of the recent advancements in understanding ferroptosis in cancer, covering molecular mechanisms, biological functions, regulatory pathways, and interactions with the tumor microenvironment. We also summarize the potential applications of ferroptosis induction in immunotherapy, radiotherapy, and systemic therapy, as well as ferroptosis inhibition for cancer treatment in various conditions. We finally discuss ferroptosis markers, the current challenges and future directions of ferroptosis in the treatment of cancer.

## Introduction

Every living being eventually dies. Cell death is a key biological process inherent in complex organisms, serving as a crucial mechanism for the elimination of unwanted cells.^1^ Mammalian cell death encompasses accidental cell death, an uncontrolled biological event triggered by unexpected attacks and injuries, and regulated cell death (RCD), which is driven by a genetically encoded apparatus and can be modulated by drug or genetic interventions.^2^ The orderly progression of RCD in complex organisms is integral to its normal development and homeostasis,^3^ while the loss of controlled cell death contributes to human diseases such as cancers, characterized by the presence of abnormal cells exhibiting unlimited replication and immortality due to successful evasion of cell death regulation. Cancer treatment strategies consistently prioritize the selective eradication of cancer cells while minimizing harm to normal cells. RCD is an important channel for achieving this, as it enables the specific targeting of tumor cells and enhances the efficacy of drug-induced cell death, while simultaneously reducing adverse effects on normal cells.

Ferroptosis, a term coined by the laboratory of Brent R. Stockwell in 2012, is a distinct mode of RCD characterized by the iron-dependent lethal accumulation of membrane-localized lipid peroxides.^4^ Cells undergoing ferroptosis display distinct hallmarks compared to other extensively studied forms of RCD,^5^ such as apoptosis,^6^ pyroptosis,^7^ and necroptosis.^5^ Morphologically, ferroptotic cells exhibit dysmorphic small mitochondria with condensed membranes and decreased crista.^4,8–10^ Mechanically, unlike classical RCD involving specific executioner proteins of cell death (such as gasdermin D for pyroptosis, caspase for apoptosis, and mixed lineage kinase domain-like protein (MLKL) for necrosis), the identity of the cell death executioner proteins in ferroptosis remains unclear. While it is widely accepted that the execution of ferroptosis necessitates the oxidized phospholipids (PLs) containing polyunsaturated fatty acids (PUFA-PLs), the mechanisms by which these oxidized PUFA-PLs, beyond a certain threshold, lead to membrane permeabilization and cell death, as well as the downstream executioners that mediate the eventual execution event known as the ‘point of no return’ in ferroptosis, remain largely elusive.^11^ The process of ferroptosis involves ferrous iron accumulation, free radical production, antioxidant system dysfunction, and lipid peroxidation. Based on its distinctive features, a comprehensive panel of biomarkers and functional tests, including pharmacological inhibition, has been assembled to effectively differentiate ferroptosis from other types of RCD, providing suitable tools for investigating the pathophysiological functions of ferroptosis.^12^

In recent years, modulating ferroptosis to intervene in the occurrence and development of cancer has been a hotspot and focus of etiological research and treatment. Ferroptosis is tightly implicated in tumor biology. On the one hand, tumor suppressors have been found to execute part of their tumor-suppression function depending on ferroptosis induction. Ferroptosis seems to be an innate tumor-suppressive mechanism.^12,13^ On the other hand, cancer cells, in order to support their survival, can evolve several mechanisms to evade host ferroptosis, which provides vulnerable targets for ferroptosis-based therapy. Interestingly, mesenchymal and dedifferentiated cancer cells, which typically exhibit resistance to apoptosis and traditional treatment approaches, display a remarkable susceptibility to ferroptosis. Consequently, ferroptosis is recognized as an attractive target for cancer treatments, especially for refractory tumors. So far, a plethora of ferroptosis interventions have shown promising effectiveness in cancer treatment even overcoming resistance to traditional therapies,^14–16^ and ferroptosis is also involved in the tumor-suppressive functions of radiotherapy and immunotherapy.^17–20^ Combination therapy based on ferroptosis is a highly promising strategy for enhancing the effectiveness of conventional therapies, tackling resistant tumors, and preventing tumor recurrence. However, the role of ferroptosis in tumor suppression depends on the context, as it appears to have apparently paradoxical roles in different stages of some tumors. For instance, ferroptosis induction facilitates the progression of chronic liver diseases to hepatocellular carcinoma (HCC),^21^ while it can restrain the established HCC development.^22^ Moreover, a study found that ferroptosis inhibitors can effectively suppress tumor growth as long as they are administered early when the tumor is sufficiently small.^23^ Therefore, achieving a comprehensive and in-depth understanding of the role of ferroptosis in cancers is crucial for effectively guiding its application in cancer treatment.

Given the vigorous growth in ferroptosis, it is imperative to gain iterative insights into ferroptosis. Here, we review the major milestones and molecular machinery of ferroptosis, including drivers and defenses two systems. Then, we decipher the functions of ferroptosis in tumor biology, the classic cancer-related ferroptosis regulatory pathways and ferroptosis-mediated crosstalk between cancers and immune cells. Lastly, we summarize the potential ferroptosis-based therapy and ferroptosis markers and discuss the current limitations and future directions of ferroptosis in the treatment of cancer.

## Major milestone of ferroptosis

**Fig. 1:**
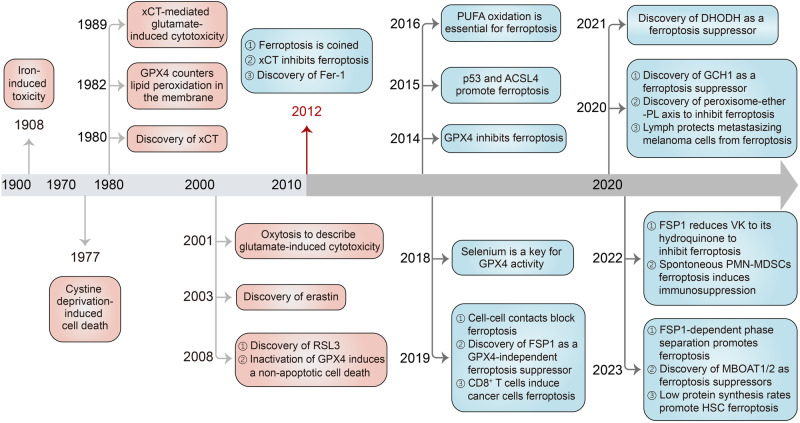
History of research on the discovery and development of ferroptosis. The term ferroptosis was coined in 2012, but the understanding of ferroptosis can be traced back as early as 1908. Since 2012, there has been a flourishing development in the research of ferroptosis and its regulatory mechanisms. ACSL4 acyl-CoA synthetase long-chain family member 4, DHODH dihydroorotate dehydrogenase, Fer-1 ferrostatin-1, FSP1 ferroptosis suppressor protein 1, GCH1 GTP cyclohydrolase 1, GPX4 glutathione peroxidase 4, HSC hematopoietic stem cells, MBOAT1/2 membrane-bound O-acyltransferase domain-containing 1 and 2, PL phospholipid, PMN-MDSC polymorphonuclear myeloid-derived suppressor cell, PUFA polyunsaturated fatty acid, VK vitamin K

In the past decade, we have witnessed a significant surge in research on ferroptosis^24^ **(**Fig. 1**)**. Although the term ferroptosis was coined in 2012,^4^ the clues to ferroptosis date back much earlier. Iron-induced toxicity was first observed in 1908.^25^ The importance of cystine in the viability and growth of mouse fibroblast strain L and the HeLa cell was reported in 1955.^26,27^ Dietary cystine and selenium in 1959 were further found to significantly reduce peroxidation in the liver and muscle of vitamin E-deficient chicks.^28^ In 1977, Shiro Bannai and colleagues observed that withdrawal of cystine-induced cell death accompanied by glutathione (GSH) depletion could be rescued by antioxidant vitamin E supplementation.^29^ They further reported in 1980 that cystine could be taken up from the environment by the system xc^-^ in exchange for glutamate.^30^ The identification of glutathione peroxidase 4 (GPX4), a selenoprotein, in 1982 as a GSH-dependent peroxidase to counteract lipid peroxidation in membranes, marked a significant milestone.^31^ Over the following decade, GPX4 was found to counteract cell death associated with lipid peroxidation.^32^ In 1989, it was observed that glutamate-induced cytotoxicity resulted from the cystine uptake inhibition, leading to decreased GSH levels, oxidative stress, and ultimate cell death.^33^ Antioxidant treatments, such as alpha-tocopherol (α-toc), as well as inhibition of iron-containing lipid dioxygenase arachidonate lipoxygenase 12 (ALOX12), in 1992 and 1997, respectively, were found to prevent this form of cell death.^34,35^ Remarkedly, in 2001, the concept of “oxytosis” was coined to characterize the non-apoptotic cell death in neurons that is induced by oxidative stress in response to glutamate toxicity.^36^ While both oxytosis and ferroptosis involve reactive oxygen species (ROS) production, ALOXs and GSH depletion,^36^ and could be suppressed by iron chelators and enhanced by various sources of iron,^37^ some special features in oxytosis including cyclic guanosine monophosphate (cGMP)-gated channels, mitochondrial swelling and DNA fragmentation,^38^ highlighted ferroptosis as a distinct form of RCD.

The discovery of ferroptosis stemmed from the high-throughput screening of small molecules aimed at targeting oncogenic RAS mutations. In 2003, erastin was identified as a selective inducer of non-apoptotic cell death in cancer cells dependent on ST- and RASG12V,^39^ along with the involvement of the RAS/BRAF/MEK/MAPK pathway and voltage-dependent anion channel (VDAC) that mediate oxidative stress and mitochondrial dysfunction, respectively.^40^ Another small molecule compound, RSL3, was discovered in 2008 through the same screening system, which could activate an iron-dependent form of cell death.^41^ In the same year, the inactivation of GPX4 was reported to induce a non-apoptotic cell death that could be suppressed by alpha-tocopherol and ALOX12/15 inhibitors.^42^ It was only in 2012 that the term “ferroptosis” was coined to describe this form of cell death, due to its dependence on iron, unique morphology, biochemical traits, and genetic features that distinguish it from other forms of regulated cell death.^4^ Erastin was discovered to block cystine uptake by inhibiting system xc^-^ to induce ferroptosis, while ferrostatin-1 was identified as a powerful inhibitor of ferroptosis in cancer cells.^4^ In the following decades, breakthroughs in ferroptosis yielded a comprehensive insight into the mechanisms responsible for the execution and regulation of this process.

In 2014, GPX4 was identified as the central regulator of RSL3- and erastin-induced ferroptosis, and RSL3 directly inactivates GPX4, leading to lipid peroxidation and ultimately ferroptosis.^43^ Moreover, knockout of GPX4 causes cell death, while liproxstatin-1, a potent spiroquinoxalinamine derivative, is reported to suppress ferroptosis.^8^ In 2015, through the extensive use of massive insertional mutagenesis on haploid KBM7 cells, the inactivation of acyl-coenzyme A (CoA) synthetase long-chain family member 4 (ACSL4) and lysophosphatidylcholine acyltransferase 3 (LPCAT3) was shown to render these cells resistant to ferroptosis. Meanwhile, the most commonly mutated tumor suppressor protein, p53, suppresses solute carrier family 7 members 11 (SLC7A11) expression and cystine uptake, sensitizing cells to ferroptosis.^13^ In 2016, ferroptosis was found to rely on PUFA oxidation by ALOXs via a phosphorylase kinase G2 (PHKG2)-dependent iron pool and the covalent inhibition of the catalytic selenocysteine in GPX4 hinders the removal of PUFA hydroperoxides.^44^ Simultaneously, it has been reported that FIN56 not only induces the degradation of GPX4 but also depletes ubiquinone (CoQ_10_) through the mevalonate pathway to enhance ferroptosis sensitivity.^45^ In 2017, ACSL4 was further identified as an essential component for ferroptosis execution by promoting arachidonic acid (AA) or adrenic acid (AdA) esterification into phosphatidylethanolamines (PEs).^46^ A further study in 2018 underlined the requirement for selenium utilization by GPX4 to inhibit hydroperoxide-induced ferroptosis.^45^ In 2019, CD8^+^ T cells were shown to induce tumor ferroptosis during cancer immunotherapy.^19^ In the meantime, E-cadherin-mediated intercellular contacts control ferroptosis sensitivity through the Merlin/Hippo/Yes-associated protein 1(YAP) pathway to regulate the expression of ACSL4 and transferrin receptor (TFR1) in response to cell-cell contacts.^47^ Moreover, using unbiased genetic screens, ferroptosis suppressor protein (FSP1), previously named apoptosis-inducing factor mitochondria-associated 2 (AIFM2), was independently discovered as a novel ferroptosis resistance gene capable of complementing the loss or inhibition of GPX4.^48,49^

Two groups in 2020 independently identified GTP cyclohydrolase-1 (GCH1) as a suppressor of ferroptosis.^50,51^ Mechanistically, GCH1 suppresses ferroptosis through two main mechanisms. First, it produces the lipophilic antioxidant tetrahydrobiopterin (BH4), which aids in the prevention of lipid peroxidation. Second, GCH1 increases the abundance of the reducing agent CoQ_10_, which further protects against ferroptosis. This dual action of GCH1 contributes to the suppression of lipid peroxidation and the maintenance of cellular redox balance.^50,51^ Moreover, Zou et al. identified the oxidative organelles peroxisomes as a crucial factor in driving susceptibility to and evasion from ferroptosis through the synthesis of polyunsaturated ether PLs (PUFA-ePLs), which serve as substrates for lipid peroxidation.^52^ The administration of the engineered enzyme cyst(e)inase demonstrated a viable method to trigger ferroptosis in pancreatic ductal adenocarcinoma (PDAC) by depleting cysteine and cystine.^53^ Additionally, Ubellacker et al. found that melanoma cells in the lymph are prone to forming metastases in blood because lymph protects metastasizing melanoma cells from ferroptosis.^54^ In 2021, dihydroorotate dehydrogenase (DHODH) was discovered to be a mitochondrial suppressor of ferroptosis through its ability to decrease mitochondrial CoQ_10_ levels, and DHODH inhibitors had ferroptosis-sensitizing effects which was argued by Mishima et al. that DHODH inhibitors enhance sensitivity to ferroptosis through the inhibition of FSP1.^55^ In 2022, it was discovered that pathologically activated neutrophils, known as polymorphonuclear (PMN) myeloid-derived suppressor cells (MDSCs), undergo spontaneous ferroptosis, which contributes to immune suppression in cancer, highlighting the role of ferroptosis in immune regulation within the tumor microenvironment.^23^ Furthermore, FSP1 was further discovered to efficiently reduce vitamin K to its hydroquinone, providing protection against harmful lipid peroxidation and ferroptosis.^56^ Further studies in 2023 demonstrated that FSP1-dependent phase separation is crucial for ferroptosis induction.^57^ Besides, Zhao et al. found that low protein synthesis rates increased the susceptibility of hematopoietic stem cells to ferroptosis.^58^ Strikingly, through a whole-genome CRISPR activation screen, sex hormone-driven membrane-bound O-acyltransferase domain-containing 1 and 2 (MBOAT1/2) expressions were reported to prevent ferroptosis in cancer cells lacking the two main ferroptosis defense systems GPX4 and FSP1.^59^ Collectively, these studies contribute to a deeper understanding of the mechanisms and regulation of ferroptosis, highlighting its significance in the potential for therapeutic interventions.

## Molecular mechanisms of ferroptosis

**Fig. 2:**
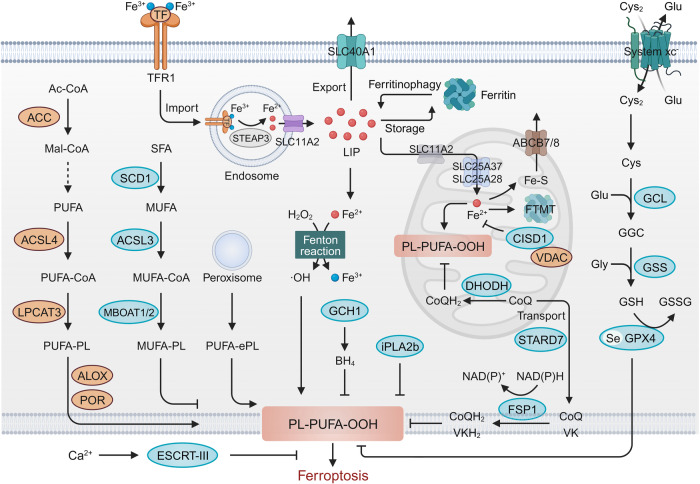
Molecular mechanisms of ferroptosis. Ferroptosis is driven by PUFA-PLs synthesis, lipid peroxidation and iron toxicity. Major defense systems of ferroptosis include the GPX4 antioxidant system, FSP1/ubiquinol (CoQH_2_), DHODH/CoQH_2_, GCH1/tetrahydrobiopterin (BH4) systems, monounsaturated fatty acid (MUFA)-PLs synthesis, and the ESCRT-III-mediated membrane repair systems. When ferroptosis-promoting activities significantly surpass the detoxification capabilities provided by the defense systems, a fatal accumulation of lipid peroxides on the cellular membranes ultimately results in membrane rupture and ferroptotic cell death. ABCB7 ATP binding cassette subfamily B member 7, ACC acetyl-CoA carboxylase, ALOX lipoxygenase, CISD1 CDGSH iron sulfur domain 1, CoQ coenzyme Q, Cys cysteine, Cys2 cystine, FTMT ferritin mitochondrial, GCL glutamate-cysteine ligase, GSH glutathione, GSSG oxidized glutathione, iPLA2b phospholipase A2 group VI, LIP labile iron pool, LPCAT3 lysophosphatidylcholine acyltransferase 3, NAD(P)H nicotinamide adenine dinucleotide phosphate, POR cytochrome P450 oxidoreductase, SCD1 stearoyl-CoA desaturase 1, SFA saturated fatty acid, SLC25A37, solute carrier family 25 member 37, SLC25A28 solute carrier family 25 member 28, SLC40A1 solute carrier family 40 member 1, STARD7 StAR-related lipid transfer domain containing 7, TF transferrin, TFR1 transferrin receptor, VDAC voltage-dependent anion channel. This figure was created with BioRender.com

Ferroptosis reflects a redox imbalance between its drivers and defenses system.^24,60^ Here, we briefly outline its core mechanisms, with a specific emphasis on its driving and defense mechanisms (Fig. 2). For a more comprehensive and detailed understanding of the molecular pathways and intricate mechanisms underlying ferroptosis, we recommend referring to several recent reviews in the field.^12,24,60–64^

### Drivers of ferroptosis

#### PUFA-PLs synthesis

PUFAs are highly prone to lipid peroxidation due to the presence of weak C-H bonds at the bis-allylic positions.^44,65^ Recent studies mainly focus on ω-6 PUFAs, such as linoleic acid (18:2), gamma-linolenic acid (18:3), dihomo-gamma-linolenic acid (20:3), AA (20:4) and AdA (22:4), as well as ω-3 PUFAs, including alpha-linolenic acid (18:3), eicosapentaenoic acid (20:5) and docosahexaenoic acid (22:6).^66,67^ Among them, AA (20:4) and AdA (22:4), are the primary substrates of lipid peroxidation during ferroptosis.^68^ Notably, free PUFAs are not the direct drivers of ferroptosis, and they need to be esterified into membrane PLs to exhibit lethality after peroxidation.^46,68,69^ ACSL4 and LPCAT3 are responsible for the biosynthesis and esterification of PUFA-PLs. Taking AA (20:4) as a case in point, ACSL4 catalyzes the combination of free AA (20:4) and CoA to form a CoA-AA (20:4) intermediate, which is subsequently esterified into PEs by LPCAT3 to generate AA (20:4)-PE (PE-AA),^46,68,69^ which are necessary for the execution of ferroptosis. Consistently, malonyl-CoA generated by acetyl-CoA carboxylase (ACC)-catalyzed carboxylation of acetyl-CoA is critical for the synthesis of certain PUFAs and is therefore necessary for ferroptosis.^60^ The peroxisome-mediated biosynthesis of plasmalogens has been suggested as an additional pathway for the production of PUFAs involved in lipid peroxidation, favoring ferroptosis onset.^52,70^ On the contrary, phospholipase A2 group VI (iPLA2β) cleaves oxidized PUFA tails from PLs to suppress p53-driven ferroptosis.^71^ Remarkedly, deuterated PUFAs (D-PUFAs) at bis-allylic position retards the radical chain reaction of lipid peroxidation to protect against RSL3- or erastin-induced ferroptosis,^44^ suggesting the significance of the structure of PUFAs in its activity.

#### Lipid peroxidation

Lipid peroxidation is the hallmark of ferroptosis.^4^ PUFA-PLs are highly susceptible to peroxidation because of the presence of bis-allylic moieties in PUFAs. The oxidation of PUFA-PLs occurs through both enzymatic reactions and non-enzymatic autoxidation driven by the Fenton reaction.^44,72^ Enzymatic lipid peroxidation of PUFA-PLs primarily involves the action of ALOXs and cytochrome P450 oxidoreductase (POR).^73^ ALOXs are enzymes containing nonheme iron, which directly introduce oxygen to PUFAs and PUFA-containing lipids within biological membranes. For example, ALOX12 is essential for p53-dependent ferroptosis, while ALOX15 is involved in erastin- or RSL3-induced ferroptosis through complexing with PE binding protein 1 (PEBP1), specifically recognizing stearoyl-AA-PE to generate lipid peroxides.^74^ Moreover, ALOXE3, ALOX5, ALOX12B, and ALOX15B have been implicated in ferroptosis induction.^75–78^ Several ALOX inhibitors have been shown to possess antioxidant properties, effectively shielding cells from lipid peroxidation.^35,42^ However, genetic deletion of Alox15 in Gpx4 knockout mice failed to avert ferroptosis in vivo,^8^ and Alox12/15 failed to restore the viability of Gpx4 deficient T cells,^79^ suggesting the existence of alternative mechanisms in certain contexts of ferroptosis. As expected, POR directly supplies electrons to the P450 enzyme, which catalyzes the peroxidation of PUFA-PLs in an ALOX-independent manner.^73,80^ These studies suggested that several iron-dependent enzymes can promote lipid peroxidation and ferroptosis. Future investigations are needed to determine the potential involvement of other oxygenases, such as cyclooxygenases and peroxygenases, in lipid peroxidation. Non-enzymatic lipid peroxidation of PUFA-PLs is driven by the Fenton reaction, with iron serving as a catalyst.^72,81^ In this process, once the initial phospholipid hydroperoxides (PLOOHs) are generated (via enzymatic reactions or other cellular metabolic processes) and are not promptly reduced by GPX4, they can interact with ferrous iron to yield alkoxyl and peroxyl radicals (Fenton reaction), initiating PLOOHs production.^82,83^

#### Iron metabolism and toxicity

As noted above, lipid peroxidation requires both iron-dependent enzymes and iron-mediated Fenton reactions, thereby at least partly providing the iron-dependent nature of ferroptosis. Thus, interventions targeting iron metabolism have an impact on the vulnerability to ferroptosis. Ferric iron is the primary form of iron in circulation and binds to transferrin (TF).^84^ It is delivered into cells and localized in endosomes with the assistance of TFR1, a membrane protein.^85^ Within the endosome, ferric iron is reduced to ferrous iron by the six-transmembrane epithelial antigen of the prostate 3 (STEAP3).^86^ The endocytosed ferrous iron is later released into the cytoplasm via solute carrier family 11 member 2 (SLC11A2), forming the labile iron pool (LIP), which catalyzes the generation of hydroxyl radicals and triggers ferroptosis.^87^ Excess intracellular iron is typically sequestered within the ferritin protein, which consists of two subunits: ferritin heavy chain 1 (FTH1) and ferritin light chain (FTL).^41^ Ferritin undergoes degradation via ferritinophagy, facilitated by nuclear receptor coactivator 4 (NCOA4), resulting in the release of substantial amounts of iron.^88,89^ Additionally, excess cytoplasmic ferrous iron can be exported from the cell through solute carrier family 40 member 1 (SLC40A1).^90^ Consistently, deletion of TF, TFR1, SLC11A2 and NCOA1, and overexpression of FTH1, FTL and SLC40A1 suppresses ferroptosis by decreasing the LIP.^91^ Therefore, interventions that modulate the import, storage, and export of iron in the cytoplasm contribute to an increase in susceptibility to ferroptosis.

In addition to the cytoplasm, mitochondria, which is the primary site of iron utilization and the main source of ROS, plays a major role in modulating redox-active reactions and ferroptosis.^92,93^ To reach the mitochondria, iron must traverse both the outer and inner mitochondrial membranes to enter the matrix through SLC11A2,^94^ and solute carrier family 25 member 37 (SLC25A37) or solute carrier family 25 member 28 (SLC25A28), respectively.^95–97^ Moreover, recent studies highlighted the key role of CDGSH iron sulfur domain 1 (CISD1) in regulating iron homeostasis in mitochondria.^98,99^ CISD1 knockdown significantly increases the content of erastin-induced mitochondrial ferrous irons and promotes mitochondrial lipid peroxidation and ferroptosis.^100^ CISD1 can also bind with VDAC proteins and regulate their gating in a redox-dependent manner.^101^ Inhibiting VDAC proteins can prevent mitoCISD1-dependent mitochondrial iron accumulation and erastin-induced ferroptosis.^40,101–103^ Ferritin mitochondrial (FTMT) serves as the iron-storage protein in mitochondria, inhibiting ferroptosis by reducing total and chelatable iron levels.^104–106^ ATP binding cassette subfamily B member 7 (ABCB7) is involved in the transfer of iron from mitochondria to cytosol.^107–109^ Although mitochondrial iron accumulation can be observed in the absence of ABCB7,^107^ ABCB7 loss does not lead to an increase in mitochondrial ROS and ferroptosis.^110^ On the contrary, ABCB8 can facilitate mitochondrial iron export.^111,112^ Overexpression of ABCB8 reduces mitochondrial iron and protects against ferroptosis-related I/R damage and doxorubicin-induced cardiomyopathy.^113,114^ Collectively, these results provide strong evidence that diverse factors controlling iron metabolism regulate susceptibility to ferroptosis.

### Defenses of ferroptosis

#### GPX4 antioxidant system

Erastin and RSL3 are the representative two types of ferroptosis inducers (FINs) through directly inhibiting the activity of xc^-^ system and GPX4, respectively.^4,43^ The system xc^-^ containing subunits SLC7A11 and solute carrier family 3 member 2 (SLC3A2) mediates the exchanges of intracellular glutamate for extracellular cystine.^115,116^ Intracellular cystine is quickly converted to cysteine, playing a vital role as a cellular antioxidant and acting as the limiting factor for the synthesis of glutamate-cysteine ligase (GCL)-mediated GSH synthesis.^117,118^ The availability of cellular GSH closely regulates the cellular GPX4 activity.^119,120^ Thus, the inactivation of GPX4 by both erastin and RSL3, either directly or indirectly, underscores the significance of GPX4 as a key repressor of ferroptosis.

GPX4 is the sole member of the GPX family that acts as a phospholipid hydroperoxidase, directly reducing PLOOH to their corresponding phospholipid alcohols (PLOH).^31,121^ GPX4’s catalytic reaction operates according to a ping-pong mechanism, where the enzyme’s active site shuttles between oxidation and reduction states. Firstly, the PLOOH oxidizes the active site selenol in GPX4 (GPX4-SeH) to form the selenenic acid intermediate (GPX4-SeOH). Secondly, this intermediate undergoes a reaction with GSH, resulting in the formation of the selenium-glutathione adduct (GPX4-Se-SG). Thirdly, through a reaction with a second GSH molecule, GPX4-Se-SG undergoes conversion to GPX4-SeH, generating oxidized glutathione (GSSG).^122,123^ By examining the crystal structure of seleno-GPX4, researchers observed the existence of seleninic acid (GPX4-Se-OO-) within the enzyme’s active site.^124^ This finding implies the possibility of an alternative reaction mechanism that encompasses three distinct redox states (GPX4-SeH, GPX4-SeOH, GPX4-Se-OO^-^) of the catalytically active selenocysteine. These studies have also emphasized the essential role of selenocysteine in the expression and activity of GPX4, which is in line with Ingold et al.’s findings that the substitution of a cysteine residue for selenocysteine (U46C) in GPX4 is required to prevent hydroperoxide-induced ferroptosis.^125^

GPX4 exists in three isoforms: mitochondrial, cytosolic, and nuclear GPX4. While derived from the same GPX4 gene, the isoforms of GPX4 have distinct transcription initiation sites.^126–129^ Early embryonic lethality occurs when the cytosolic GPX4 gene is genetically ablated or expresses an inactive form.^130^ The rescue of the lethal phenotype in Gpx4-null mutant mice was achieved by re-expression of cytoplasmic GPX4, rather than mitochondrial or nuclear GPX4, indicating the crucial role of cytosolic GPX4 in preventing embryonic lethality.^131,132^ Disruption of mitochondrial GPX4 in mice does not result in lethality but instead causes male infertility due to abnormal sperm development.^130,133^ It seems that only cytosolic GPX4 could suppress ferroptosis, which is challenged by recent studies that mitochondrial GPX4, but not cytoplasmic GPX4, could potently suppress lipid peroxidation and ferroptosis in DHODH or glycerol-3-phosphate dehydrogenase 2 (GDP2) knockout cells.^134,135^ Therefore, these organelle-specific forms of GPX4 may independently inhibit local lipid hydroperoxides and ferroptosis, although the potential role of nuclear GPX4 requires further investigation. Notably, GPX4 depletion also mediates apoptosis, necroptosis and pyroptosis in mice, suggesting that GPX4 depletion-induced lipid peroxidation occupies a central position at the intersection of these forms of RCD. Thus, the detection of multiple markers is essential for definitively identifying ferroptosis in addition to lipid peroxidation.

#### Radical-trapping antioxidant system

As mentioned above, GPX4 is a central suppressor of ferroptosis, and other mechanisms that regulate the activity or expression of GPX4 also control susceptibility to ferroptosis.^43,45,136–138^ However, some cancer cells survived GPX4 inhibition, suggesting the existence of alternative mechanisms of ferroptosis resistance. In recent years, three GPX4-independent systems that capture free radicals to exert their antioxidative effects and suppress ferroptosis have been identified. These systems include FSP1/ ubiquinol (CoQH_2_), DHODH/CoQH_2_, and GCH1/BH4. Coenzyme Q (CoQ) is an endogenous antioxidant and exists in three forms: CoQ_10_, semiquinone, and CoQH_2_, wherein CoQH_2_ traps lipid peroxyl radicals to protect cells from ferroptosis.^15,49^ The synthesis and cellular distribution of CoQ_10_ are linked to StAR related lipid transfer domain containing 7 (STARD7), which transports CoQ_10_ from mitochondria, where it is synthesized, to the plasma membrane.^139^ In 2020, two independent teams found that N-myristylation-dependent recruitment of FSP1 to the plasma membrane resists ferroptosis in the plasma membrane.^48,49,140^ Mechanistically, FSP1 suppresses lipid peroxidation by catalyzing the reduction of CoQ_10_ to CoQH_2_ with the consumption of nicotinamide adenine dinucleotide phosphate (NAD(P)H).^48^ Moreover, FSP1 was identified as a vitamin K reductase to generate its associated hydroquinone, which inhibits lipid peroxidation at the expense of NAD(P)H.^56^ A recent study also suggested that phase separation of FSP1 plays a role in promoting ferroptosis which requires N-terminal myristoylation, as well as specific amino acid residues and essentially disordered, low-complexity regions in FSP1.^57^ Analogous to the function of FSP1 in the plasma membranes, DHODH detoxifies lipid peroxides by reducing CoQ_10_ to CoQH_2_, thereby inhibiting ferroptosis specifically in the mitochondria.^134^ Furthermore, GCH1 has been identified as a suppressor of ferroptosis through a two-pronged mechanism.^48,50^ On one hand, GCH1 produces the lipophilic antioxidant BH4 preventing lipid peroxidation; on the other hand, GCH1 induces lipid remodeling as a protective measure against ferroptosis by selectively safeguarding PLs with two polyunsaturated fatty acyl tails from depletion.^48,50^ However, the subcellular compartments wherein the GCH1/BH4 system functions still need further investigation.

#### MUFA-PLs synthesis

Different from PUFAs, monounsaturated fatty acids (MUFAs) are less susceptible to peroxidation owing to a lack of bis-allylic positions. Exogenous MUFAs can prevent ferroptosis by displacing PUFAs from membrane lipids.^44,141^ The biosynthesis of anti-ferroptosis MUFA-PLs is mainly regulated by stearoyl-CoA desaturase 1 (SCD1) and acyl-CoA synthetase long-chain family member 3 (ACSL3).^141,142^ SCD1 introduces a double bond in the cis-Δ9 position of the de novo synthesized saturated fatty acids (SFAs), particularly palmitic acid (C16:0) and stearic acid (C18:0), resulting in the formation of palmitoleic acid (C16:1) and oleic acid (C18:1), respectively.^142^ As a result, overexpression of SCD1 enhances MUFA synthesis and protects cells from ferroptosis, while inhibition of SCD1 enhances the sensitivity to ferroptosis.^59,142^ Moreover, MUFAs are reported to enhance both the number of lipid droplets and the number/function of peroxisomes, leading to a reduction in ether lipids and lipid oxidation.^143^ However, oleic acid does not lower the propensity of cells to succumb to ferroptosis in ACSL3-depleted cells.^54^ ACSL3 converts MUFAs into their acyl-CoA esters, facilitating their incorporation into membrane PLs.^141^ Thus, similar to PUFAs, MUFAs need to be inserted into the membrane to exhibit antioxidant properties. Interestingly, MBOAT1/2 has recently been identified to selectively transfer MUFAs into lyso-PE, resulting in an increase in cellular PE-MUFA and a corresponding decrease in cellular PE-PUFA, ultimately resisting ferroptosis.^59^ On all accounts, the anti-ferroptosis role of MBOAT1/2 operates independently of GPX4 and FSP1 through a surveillance mechanism mediated by PL remodeling.

#### Membrane repair system

The rupture of the plasma membrane is involved at the terminal stage of ferroptosis. Membrane damages in ferroptosis cover a loss of plasma membrane integrity,^88^ and rupture of the outer mitochondria membrane.^4,8^ Consequently, membrane repair systems have been proposed and demonstrated to prevent ferroptosis. Among these systems, the Endosomal Sorting Complex Required for Transport-III (ESCRT-III) has gained attention as a common mechanism for membrane repair, acting as a defense against various forms of RCD, including ferroptosis.^144–146^ Mechanistically, ferroptosis leads to an elevation in cytosolic Ca^2+^ levels due to an osmotic imbalance triggered by the opening of small nanopores.^147^ In response to the influx of Ca,^2+^ subunits of ESCRT-III known as charged multivesicular body proteins (CHMPs), specifically CHMP5 and CHMP6, are recruited and assembled at the location of damage to facilitate membrane repair processes. Erastin and RSL3, which are known as ferroptosis activators, lead to the buildup of CHMP5 and CHMP6 in the plasma membrane of pancreatic cancer cells, and the knocking out of CHMP5 or CHMP6 intensifies the susceptibility of cancer cells to ferroptosis.^146^ Furthermore, in certain cases, FSP1 inhibits ferroptosis by promoting the accumulation of CHMP5 and CHMP6 on the plasma membrane.^148^ Overall, these findings highlight the critical role of ESCRT-III activation in preventing ferroptosis.

## Functions of ferroptosis in cancer biology

Oxygen (O_2_)-driven metabolism is vital for the survival of organisms and the execution of biological activities, achieved via a sequence of redox reactions.^149^ The transition metal iron is the key element to catalyze these redox processes, leading to the generation of ROS, which encompasses various oxygen derivatives, including ferroptosis divers PLOOHs. Accumulating evidence indicates ferroptosis in tumor biology.

### Ferroptosis induction in tumor suppression

Ferroptosis appears to function as an innate mechanism for tumor suppression, mediating the anticancer activity of several tumor suppressor genes. Tumor suppressors such as p53, BRCA1-associated protein 1 (BAP1), fumarate hydratase (FH), Kelch-like ECH-associated protein 1 (KEAP1), and the epigenetic regulator MLL4 have been shown to exert their tumor-suppressive functions, at least partially, by inducing ferroptosis in tumor cells.

The tumor suppressor TP53, widely regarded as the most critical barrier to cancer development, effectively exerts its ferroptosis-mediated tumor-suppression function by suppressing the cystine transporter SLC7A11 in an ALOX12-dependent manner.^13,150,151^ While the acetylation-defective mutant p53^3KR (K117R, K161R, K162R)^ loses its conventional functions of promoting cell-cycle arrest, apoptosis, and senescence, it still retains its tumor-suppressive ability by promoting ferroptosis.^13^ By contrast, the mutant p53^4KR (K98R+3KR)^ lack ferroptosis regulatory activity and consequently lose their tumor-suppressive functions,^152,153^ suggesting the significance of acetylation in ferroptosis. Moreover, the TP53 single-nucleotide polymorphism P47S found in many people of pre-menopausal African-American women has an increased risk of breast cancer.^152,154^ Mechanistically, p53^P47S^ is defective in promoting ferroptosis and repressing tumor development through increasing the cellular levels of CoA and GSH.^154^ These findings indicate that ferroptosis is at least partly responsible for TP53-mediated tumor suppression.

BAP1 encodes a deubiquitinase responsible for removing ubiquitin from histone 2A and frequently exhibits inactivating mutations and deletions in various sporadic cancers.^155^ Interestingly, BAP1 suppresses tumorigenesis partly through ferroptosis by repressing SLC7A11 via reducing histone 2A ubiquitination (H2Aub) occupancy on the SLC7A11 promoter.^156^ Deletions and mutations of BAP1 result in the loss of its ability to repress SLC7A11, enabling cells to evade ferroptosis and promoting tumor formation.^156^ BAP1 re-expression in a BAP1-deficient background significantly inhibited tumor development with condensed mitochondria and increased 4-hydroxynonenal (4-HNE) protein expression, which could be partially restored by the ferroptosis inhibitor liproxstatin-1,^156^ suggesting that ferroptosis at least partly contribute to BAP1’s tumor suppression in vivo.

FH is an enzyme involved in the tricarboxylic acid (TCA) cycle, which has been confirmed as a bona fide tumor suppressor in renal cancer.^157^ Genetic mutation of FH has been detected in both benign and malignant renal cancer lesions.^157–159^ Notably, renal cancer cells with FH mutations display resistance to ferroptosis and maintain their viability and ability to proliferate even when deprived of cystine. In contrast, wild-type FH cancer cells are unable to proliferate under these conditions.^160^ These findings confer the tumorigenic advantage of the loss of FH function under oxidative stress through suppressing ferroptosis, supporting the notion that ferroptosis may serve as a physiologically relevant mechanism to suppress tumors.

KEAP1, a ubiquitinated enzyme, is commonly mutated or inactivated in lung cancers.^161,162^ KEAP1 binds to nuclear factor erythroid 2-related factor 2 (NRF2) and triggers its proteasomal degradation, thereby inhibiting tumor development.^163^ Loss of KEAP1 function leads to increased tumor burden and accelerates tumor growth,^163,164^ because its mutants or deficiency in lung cancers upregulate the expression of FSP1 by stabilizing NRF2 proteins, resulting in ferroptosis resistance.^165^ Moreover, KEAP1 knockdown protects glioma cells from ferroptosis and promotes their proliferation by upregulating NRF2-mediated expression of SLC7A11.^166^ These findings indicate that the ferroptosis-promoting role of KEAP1 potentially at least partly accounts for its tumor-suppressive function.

The epigenetic regulator MLL4 is one of the most commonly mutated genes in cancer biology.^167,168^ It can activate key ALOXs genes, such as ALOX12, promoting epidermal differentiation and barrier formation and, in turn, inhibiting cutaneous squamous cell carcinomas through ferroptosis.^169^ Epidermal MLL4 deficiency results in impaired skin differentiation, the development of precancerous neoplasms and resistance to ferroptosis, accompanied by downregulation of pro-ferroptosis genes ALOXs (ALOX12, ALOX12B, and ALOXE3) and the upregulation of anti-ferroptosis genes (GPX4, SLC7A11, and SCD1).^169^ This suggests that MLL4-mediated ferroptosis serves as a critical natural mechanism in promoting epidermal differentiation, maintaining skin homeostasis, and preventing cutaneous carcinomas formation.

### Ferroptosis evasion in tumor progression

Despite the presence of the ferroptosis-mediated tumor suppression mechanism, tumors inevitably arise and progress uncontrollably, indicating the existence of the mechanism of ferroptosis evasion in tumor. Building upon the core driver and defense mechanism of ferroptosis. We will briefly discuss the mechanisms through which tumor cells have evolved to evade ferroptosis and support tumor development.

Tumor cells exhibit heightened antioxidant capacity as an adaptive response to increased levels of ROS caused by metabolic and signaling abnormalities.^170^ Stabilizing and overexpressing the anti-ferroptotic systems are crucial mechanisms evolved by tumor cells to avert ferroptosis and promote tumor progression. The upregulation of the SLC7A11/GSH/GPX4 axis, a key ferroptosis defense system, is a significant evasion mechanism evolved by tumor cells. SLC7A11 is overexpressed in multiple cancers, and it is one of the extensively studied mechanisms by which tumor cells evade ferroptosis.^53,171,172^ For instance, its upregulation by the inactivation of tumor suppressors like TP53, BAP1, and ARF confers ferroptosis evasion and promotes tumor growth.^13,156,173^ Moreover, Oncogenic KRAS activation has also been shown to upregulate SLC7A11 expression, defending against ferroptosis and promoting lung adenocarcinoma (LUAD) development.^174^ GSH, an antioxidant that functions as the cofactor of GPX4, is frequently elevated in tumors, accelerating tumor progression and therapy resistance.^170,175–179^ GPX4, the essential antioxidant peroxidase of ferroptosis, has also been found to be highly expressed in various tumors.^180,181^ Several cancer phenotypes characterized by stem cell-like or dedifferentiated states exhibit highly dependent GPX4 for survival, indicating its crucial role in evading ferroptosis and supporting tumor cell surviva.^15,182^ The radical-trapping antioxidant system mechanisms of ferroptosis, which are mediated by the FSP1 and GCH1, are also upregulated in some cancers and contribute to ferroptosis evasion and tumor development.^50,165^ Additionally, NRF2, a master regulator of antioxidant defense, which is upregulated in multiple cancers and is considered a driver of cancer progression, metastasis, and therapy resistance,^183^ regulates components of the ferroptosis cascade, including SLC7A11, GPX4 and FSP1, to defend against ferroptosis contribute to tumor progression and therapy resistance.^165,184–188^

Tumor cells also employ mechanisms that limit pro-ferroptotic systems to evade ferroptosis. Downregulation of peroxidized PUFA-PLs and reduction of the LIP within cancer cells have been associated with ferroptosis evasion and tumor progression.^189^ For instance, iPLA2β, which cleaves and detoxifies peroxidized lipids to avert ferroptosis, is overexpressed in some human cancers and is involved in inhibiting p53-mediated ferroptosis and tumor suppression.^190^ Sterol regulatory element-binding protein 2 (SREBP2)-driven iron homeostatic pathways are overexpressed in melanoma circulating tumor cells, reducing intracellular iron pools and conferring resistance to ferroptosis, contributing to cancer progression, metastasis, and drug resistance.^191^ Proteins involved in the iron-sulfur clusters (ISCs) synthesis and assembly, such as NFS1, Frataxin, and CISD2, have been found to be highly expressed in tumors, enabling cancer cells to evade ferroptosis and contribute to tumor progression by reducing the LIP.^192–194^ Breast cancer cells detaching from the extracellular matrix increase the expression of prominin 2, stimulating iron export and reducing the LIP to evade ferroptosis.^195^

Strikingly, tumor microenvironment functions in ferroptosis evasion in tumor progression. For example, the lymphatic environment, which contains an abundant amount of oleic acid, can enhance the synthesis of MUFA-phospholipids (MUFA-PLs) in melanoma cells through an ACSL3-dependent pathway, leading to resistance to oxidative stress and ferroptosis. This facilitates the migration and survival of cancer cells in lymphatics and enhances their ability to survive during subsequent metastasis via the bloodstream.^54^ Moreover, mammary adipocytes provide protection for triple-negative breast cancer (TNBC) cells against ferroptosis by secreting oleic acid,^196^ providing a unique micro-environment for cancer cell survival. Additionally, sex hormones could regulate ferroptosis surveillance.^59^ MBOAT1 and MBOAT2 could be regulated by estrogen receptors (ER) and androgen receptors (AR), respectively. Both of them could catalyze the incorporation of MUFAs into PL to mediate the ferroptosis defense mechanism independently of GPX4 and the radical-trapping antioxidant system, suggesting the potential role of ferroptosis suppression in specific contexts related to sex hormone signaling. These findings also indicate that sex differences need to be considered, and ER or AR antagonists should combine with FINs to inhibit the ER+ cancer or AR+ prostate tumor growth, respectively.^59,197^

## Cancer-related pathway in ferroptosis

### RAS signaling

**Fig. 3:**
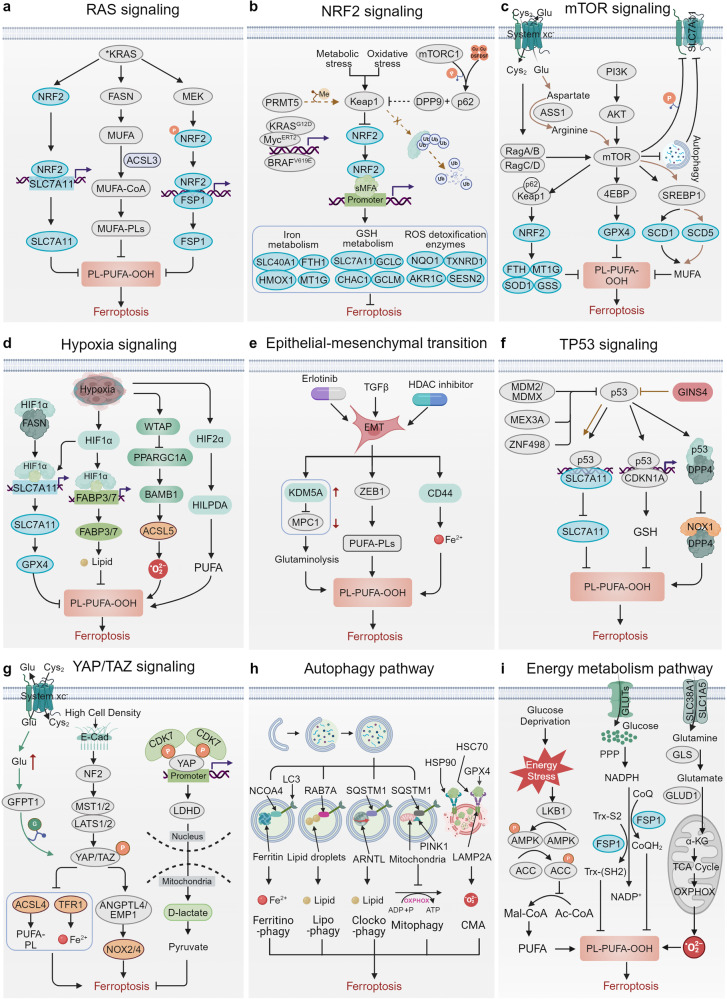
Cancer-related pathways in ferroptosis. **a** RAS signaling governs upregulation of SCL7A11, FASN, and FSP1 to evade ferroptosis, establishing a targetable vulnerability. **b** NRF2 protects cancer cells from ferroptosis primarily through transcriptional regulation of downstream target genes involved in iron metabolism, GSH metabolism and ROS detoxification enzymes. **c** mTOR signaling primarily inhibits the sensitivity to ferroptosis through autophagy, promoting GPX4 protein synthesis, and upregulating the SREBP1/SCD and KEAP1/NRF2 axis. **d** Hypoxia plays a dual role in regulating ferroptosis by inducing the expression of its primary regulators HIF1α and HIF2α. **e** EMT reshapes the metabolic status granting mesenchymal tumor cells vulnerability to ferroptosis. **f** p53 transcriptionally suppresses SLC7A11 expression or modulates metabolism-related genes to promote ferroptosis. **g** The YAP/TAZ pathway plays a crucial role in regulating cell density-mediated and D-lactate-induced ferroptosis. **h** Ferroptosis serves as a type of autophagy-dependent cell death involving ferritinophagy, lipophagy, mitophagy, clockophagy, and chaperone-mediated autophagy. **i** Mitochondrial TCA cycle, ETC and glutamate are required for cystine deprivation-induced ferroptosis. PPP generate NADPH to implicate in ferroptosis process. Energy stresses facilitate tumor defense against ferroptosis by activating AMPK to enhance ACC-mediated MUFA formation. 4EBP 4E (eIF4E)-binding proteins, α-KG α-Ketoglutaric acid, ACSL5 acyl-CoA synthetase long chain family member 5, AKT AKT serine/threonine kinase, ASS1 argininosuccinate synthase 1, AKR1C1 aldo-keto reductase family 1 member C1, ANGPTL4 angiopoietin-like 4, ARNTL aryl hydrocarbon receptor nuclear translocator like, AMPK protein kinase AMP-activated catalytic subunit alpha 1, ATM ataxia-telangiectasia mutated, BAMBI BMP and activin membrane bound inhibitor, BRAF B-Raf proto-oncogene, serine/threonine kinase, CDKN1A cyclin dependent kinase inhibitor 1A, CDK7 cyclin dependent kinase 7, CHAC1 ChaC glutathione specific gamma-glutamylcyclotransferase 1, DPP4 dipeptidyl peptidase 4, DPP9 dipeptidyl peptidase 9, EGLN2 egl-9 family hypoxia inducible factor 2, EMP1 epithelial membrane protein 1, EMT epithelial-mesenchymal transition, FABP3/7 fatty acid binding protein 3/7, FASN fatty acid synthase, FTH1 ferritin heavy chain 1, GCLC glutamate-cysteine ligase catalytic subunit, GCLM glutamate-cysteine ligase modifier subunit, GFPT1 glutamine--fructose-6-phosphate transaminase 1, GINS4 GINS complex subunit 4, GLS glutaminase, GLUD1 glutamate dehydrogenase 1, HDAC Type-2 histone deacetylase 2, HIF1α hypoxia inducible factor 1 subunit alpha, HIF2α hypoxia inducible factor 2 subunit alpha, HILPDA hypoxia inducible lipid droplet associated, HMOX1 heme oxygenase 1, HSP90 heat shock protein 90, HSC70 heat shock cognate 71 kDa protein, Keap1 Kelch-1ike ECH- associated protein l, KDM5A lysine demethylase 5A, *KRAS mutant KRAS, KRAS, KRAS proto-oncogene, GTPase, LATS1 large tumor suppressor kinase 1, LAMP2A lysosomal-associated membrane protein 2, LC3 MAP1LC3A microtubule associated protein 1 light chain 3 alpha, LDHD lactate dehydrogenase D, LKB1 Lkb1 kinase, MEK MAP kinase-ERK kinase, MDM2 proto-oncogene, MDMX MDM4 regulator of p53, MEX3A mex-3 RNA binding family member A, mTOR rapamycin target protein, MT1G metallothionein 1G, MPC1 mitochondrial pyruvate carrier 1, MST macrophage stimulating, MYC MYC proto-oncogene, bHLH transcription factor, NCOA4 nuclear receptor coactivator 4, NRF2 nuclear factor erythroid 2-related factor 2, NF2 neurofibromin 2, NOX2 NADPH oxidase 2, NOX4 NADPH oxidase 4, OXPHOX oxidative phosphorylation, PI3K phosphoinositide 3-kinase, PPARGC1A PPARG coactivator 1 alpha, PRMT5 protein arginine methyltransferase 5, RAB7A member RAS oncogene family, SCD5 stearoyl-Coenzyme A desaturase 5, SREBP1 sterol regulatory element-binding protein 1, SESN2 sestrin 2, E-cad E-cadherin, SLC40A1 solute carrier family 40 member 1, SLC7A11 solute carrier family 7 member 11, SOD1 superoxide dismutase 1, SQSTM1 sequestosome 1, TAZ Tafazzin, TXNRD1 thioredoxin reductase 1, TCA cycle tricarboxylic acid cycle, WTAP WT1 associated protein, YAP1 Yes1 associated transcriptional regulator, ZEB1 zinc finger E-box binding homeobox 1, ZNF498 zinc finger and SCAN domain containing 25. This figure was created with BioRender.com

The RAS family are the most frequently mutated oncogenes in human cancers, comprising three major mutation variants: KRAS, HRAS, and NRAS.^198,199^ RAS is the first oncogene associated with ferroptosis, due to erastin and RSL3 were initially discovered from RAS synthetic lethal screen.^39–41^ Inhibiting RAS or the downstream RAF/MEK/MAPK axis can reverse the erastin or RSL3-induced selective cytotoxicity in engineered RAS-mutant tumor cells, possibly because mutant RAS signaling enhances the cellular basal iron by modulating the expression of iron metabolism-related genes.^40,41^ Mutations in epidermal growth factor receptor (EGFR), the RAS signaling upstream, increase the sensitivity of ferroptosis in non-small cell lung cancer (NSCLC) cells and human mammary epithelial cells.^200^ Notably, mutant KRAS has been reported to evade ferroptosis, establishing a targetable vulnerability in KRAS-mutant lung cancer^174,201^ **(**Fig. 3a**)**. Mutant KRAS upregulate the NRF2/SLC7A11 axis, resulting in the selective SLC7A11 inhibition killing in KRAS-mutant cancer cells.^174^ Mutant KRAS can also elevate FSP1 by activating MAPK and NRF2 pathways to protect KRAS-mutant cells from ferroptosis during tumor initiation.^202^ Combining FSP1 in ferroptosis-inducing therapy represents an effective strategy for treating KRAS-mutant tumors.^202^ Moreover, mutant KRAS lung cancer has been found to upregulate fatty acid synthase (FASN) and escape from ferroptosis by promoting the synthesis and availability of SFA/MUFA, potentially through an ACSL3-dependent mechanism.^201^ ACSL3, downstream of FASN, is essential for tumorigenesis in mutant KRAS lung cancer.^203^ Targeting FASN represents an effective therapeutic strategy for inducing ferroptosis in mutant KRAS lung cancer.

### NRF2 signaling

NRF2 is a crucial transcription factor involved in cellular defense against oxidative and electrophilic stress.^204^ NRF2 acts as a suppressor of tumor initiation in the early stages of cancer.^205–207^ However, once oncogenic driver mutations occur, the high expression status of NRF2 in cancer cells may promote tumor progression and therapeutic resistance,^208,209^ partly through its ability to defend against ferroptosis. NRF2 primarily defends against ferroptosis through the transcriptional regulation of downstream target genes involved in iron metabolism (including SLC40A1, metallothionein 1G (MT1G), heme oxygenase 1 (HMOX1), and FTH1), GSH metabolism (including SLC7A11, glutamate-cysteine ligase catalytic subunit (GCLC), glutamate-cysteine ligase modifier subunit (GCLM), and ChaC glutathione-specific gamma-glutamylcyclotransferase 1 (CHAC1)) and ROS detoxification enzymes (including thioredoxin reductase 1 (TXNRD1), aldo-keto reductase family 1 member C 1/2/3 (AKR1C1/2/3), sestrin 2 (SESN2), glutathione S-transferase pi 1 (GSTP1), and NAD(P)H quinone dehydrogenase 1(NQO1)), thus suppressing oxidative damage induced by ferroptotic stress^210,211^ **(**Fig. 3b**)**. This transcriptional regulation mechanism relies heavily on the stability of NRF2, which is negatively regulated by the ubiquitin ligase scaffold protein KEAP1, a tumor suppressor frequently mutated in NSCLC, via the ubiquitin-proteasome pathway.^212^ Protein arginine methyltransferase 5 (PRMT5) inhibits NRF2 by methylating and stabilizing KEAP1.^213^ p62 and dipeptidyl peptidase 9 (DPP9) disrupt the interaction between KEAP1 and NRF2 by competitively binding to KEAP1 to maintain the NRF2 stability and promote the transcription of its downstream iron metabolism and antioxidant genes, resulting in ferroptosis-mediated sorafenib resistance.^187,214^ mTORC1 and disulfiram/copper (DSF/Cu)-activated p62 phosphorylation, along with mitochondrial translocator protein (TSPO)-mediated p62 accumulation by inhibiting its autophagy, enhance the competitive inhibition of p62 on KEAP1, resulting in increased NRF2 accumulation and ferroptosis resistance.^215–217^ In addition to KEAP1-mediated degradation, other oncogenes such as KRAS^G12D^, BRAF^V619E^ and Myc^ERT2^ can transcriptionally induce NRF2 expression, ensuring the maintenance of stable cellular antioxidant programs to reduce intracellular ROS accumulation and potentially provide defense against ferroptosis.^208^ Nevertheless, a recent study has revealed that the regulatory function of NRF2 in ferroptosis is influenced by cellular ferrous ions in cancer cells.^213^ Overexpression of NRF2 can promote RSL3-induced cell death in TNBC cells, which harbor high levels of ferrous ions.^213^ Further research is needed to investigate the role of NRF2 in mediating ferroptosis under different conditions.

### mTOR signaling

The mammalian target of rapamycin (mTOR), a serine/threonine protein kinase,^218^ is a key target in cancer research due to its involvement in the PI3K/AKT/mTOR signaling pathway, which is frequently activated in human cancers and is often associated with therapeutic resistance.^219^ Both mTORC1 and mTORC2 have been implicated in ferroptosis in human cancers. mTORC2 inhibits the cystine-glutamate reverse transport activity and promotes ferroptosis by phosphorylating serine at position 26 of SLC7A11.^20^ Nevertheless, mTORC1 primarily inhibits ferroptosis sensitivity through three mechanisms (Fig. 3c): inhibition of autophagy, promotion of GPX4 protein synthesis, and upregulation of the sterol regulatory element-binding protein 1 (SREBP1)/SCD axis.^220^ mTORC1 acts as a potent autophagy inhibitor via the phosphorylation-dependent inhibition of autophagy-related gene (ATG) complexes. Large tumor suppressor 1/2 (LATS1/2) kinases, core components of the Hippo pathway, are activated under high cell density conditions, leading to mTORC1 phosphorylation and subsequent inhibition of autophagy-induced degradation of SLC7A11, ultimately suppressing ferroptosis.^221^ Cysteine, mediated by SLC7A11, participates not only in GSH biosynthesis but also activates Rag/mTORC1/eukaryotic initiation factor 4E (eIF4E)-binding proteins (4EBPs) signaling pathway to promote GPX4 protein synthesis, revealing a novel mechanism of ferroptosis resistance through GPX4 metabolism.^222^ Oncogenic mutations in the PI3K/AKT pathway activate mTORC1, but not mTORC2, to promote SREBP1 expression, which in turn induces SCD1-mediated MUFAs synthesis and inhibit ferroptosis.^217^ We also reported that lorlatinib sensitizes ferroptosis by inhibiting PI3K/AKT/mTOR-mediated SREBP1/SCD1 signaling axis via targeting insulin-like growth factor 1 receptor (IGF1R) and synergizes with RSL3 to inhibit melanoma.^223^ Argininosuccinate synthase 1 (ASS1), a key enzyme in the urea cycle, can activate the mTORC1/SREBP1/SCD5 signaling pathway to promote the synthesis of MUFAs, thereby suppressing ferroptosis.^224^ Notably, mTORC1 can modulate the KEAP1/NRF2 signaling pathway by promoting the binding of p62 and KEAP1, indicating a crosstalk between the PI3K/AKT/mTOR and the KEAP1/NRF2 signaling pathways.^217^

### Hypoxia signaling

Hypoxia, a common characteristic of cancer, is present in approximately 90% of solid tumors, and it promotes tumor progression and therapy resistance.^225–227^ The hypoxic response is mainly mediated by hypoxia-inducible factors (HIFs) that are widely upregulated in human cancers and play a critical role in enabling cancer cells to adapt to hypoxic environments.^228–230^ HIFs seem to play a dual role in modulating ferroptosis and subsequently affecting therapeutic efficacy in cancers (Fig. 3d). In human fibrosarcoma and lung cancer cells, hypoxia pretreatment has been demonstrated to limit RSL3/FIN56-induced ferroptosis by inducing HIF1α expression.^231^ Mechanistically, hypoxia-induced HIF1α expression transcriptionally upregulates fatty acid-binding proteins 3 and 7 (FABP3/7), promoting lipid droplet formation via enhancing fatty acid uptake and lipid storage to evade ferroptosis.^231^ Hypoxia can enhance intracellular lactate accumulation and increase cystine uptake by promoting HIF1α-mediated transcription of lactate dehydrogenases (LDH) and SLC7A11, ultimately promoting resistance to ferroptosis in solid tumors in a lactate/GPX4-dependent manner.^232^ Additionally, under hypoxic conditions, WTAP-mediated m6A modification modulates the PPARGC1A/BAMBI/ACSL5 axis, suppressing ROS production and subsequent lipid peroxidation to inhibit ferroptosis.^233^ FASN, which is significantly upregulated in cancers with treatment-resistant features, can bind to HIF1α and inhibit its ubiquitination and degradation, facilitating the nuclear translocation of HIF1α and subsequently promoting the transcription of SLC7A11, leading to resistance to ferroptosis and sorafenib treatment in HCC.^234^ Therefore, inhibiting hypoxia-activated HIF1α signaling may be an effective strategy to reverse drug resistance by enhancing ferroptosis. Notably, hypoxia also can confer ferroptosis susceptibility to colorectal cancer cells by increasing the expression of lipid and iron-regulatory genes in a HIF2α-dependent manner.^235^ Similarly, the activation of HIF2α increases hypoxia-inducible lipid droplet-associated protein (HILPDA) expression, driving the accumulation of PUFAs and subsequent lipid peroxidation, which contributes to the vulnerability of clear-cell carcinomas to ferroptosis.^236^ This evidence reveals the complex mechanisms through which hypoxia regulates ferroptosis in cancer and emphasizes the importance of targeting hypoxia signaling as a crucial approach in anticancer therapies.

### Epithelial-mesenchymal transition

Epithelial-mesenchymal transition (EMT) is a major driver of cancer progression, as it involves the reorganization of cellular cytoskeleton, acquisition of mesenchymal features, and distant metastasis.^237–240^ Notably, EMT not only promotes the colonization of tumor cells in distant sites through a metastatic cascade but also renders these mesenchymal-like cells resistant to multiple treatment strategies.^241–243^ Interestingly, tumor cells with mesenchymal characteristics are more sensitive to ferroptosis compared to epithelial cells, partly due to the upregulation of zinc finger E-box binding homeobox 1 (ZEB1).^20,240,244,245^ ZEB1 is an EMT-related transcription factor that promotes the maintenance of mesenchymal phenotype which can be induced by TGFβ.^246^ ZEB1 enhance PUFA-PLs accumulation partially via direct transcriptional activation of the lipid biology regulator peroxisome proliferator-activated receptor gamma (PPARG), endowing susceptibility to ferroptosis.^247^ (Fig. 3e). Moreover, iron metabolism reprogramming may also contribute to the ferroptosis vulnerability of mesenchymal cells. CD44-mediated hyaluronate-dependent iron endocytosis pathway is enhanced during EMT. Endocytosed iron acts as a catalyst to relieve epigenetic suppression of mesenchymal-related proteins, thereby sustaining cellular mesenchymal characteristics and supporting ferroptosis vulnerability.^248^ This finding reveals the connection between epigenetic regulation of EMT and ferroptosis vulnerability. Notably, EMT could be induced by histone deacetylase inhibitor with increased intracellular iron accumulation and reduced expression of the iron export protein ferroportin, thereby enhancing vulnerability to ferroptosis.^249^ Erlotinib-tolerant persistent cancer cells also maintain mesenchymal characteristics with increased glutaminolysis induced by histone lysine demethylase 5 A (KDM5A) mediated mitochondrial pyruvate carrier 1 (MPC1) inhibition.^250^ Consequently, these cells become susceptible to ferroptosis. These findings shed light on the potential to selectively eliminate multidrug-resistant cancer cells with mesenchymal-like phenotypes using ferroptosis-inducing drugs, which may lay the foundation for significant advances in the field of cancer therapy resistance.

### TP53 signaling

As noted above, P53 mediates tumor suppression partly through SLC7A11 inhibition-induced ferroptosis^13,150,152–154^ (Fig. 3f). Consistently, cell cycle promoter GINS4 suppresses ferroptosis in LUAD via inhibiting p53 acetylation and promoting SLC7A11 expression.^251^ MDM2/MDMX, MEX3A, and ZNF498 inhibit p53’s transcriptional activity through post-translational modifications in different subtype cancer cells, thereby suppressing p53-mediated ferroptosis.^252–254^ However, p53 can also inhibit ferroptosis in a context-dependent manner. Upon cystine deprivation, p53 induces cyclin-dependent kinase inhibitor 1 A (CDKN1A)/p21 expression and reduces the ferroptosis sensitivity of tumor cells in a GSH-dependent manner by affecting cysteine metabolism.^255,256^ Additionally, p53 directly binds to dipeptidyl-peptidase-4 (DPP4), blocking its activity, thus inhibiting DPP4/ NADPH oxidase 1 (NOX1) complex-mediated lipid peroxidation and erastin-induced ferroptosis.^257^ Notably, TP53 null cancer cells can still undergo ferroptosis via p53-independent pathways,^173,258^ which may indicate the potential limitation of p53 as a regulator of ferroptosis.

### YAP/TAZ signaling

Cancer cells show density-dependent vulnerability to ferroptosis, with increased resistance observed in spheroids,^47^ suggesting the impact of cell density and cell-cell connections on ferroptosis sensitivity independent of genetic factors. The Hippo pathway, the primary regulator of intercellular communication and mechanical forces, plays a critical role in modulating density-mediated ferroptosis susceptibility.^259^ (Fig. 3g). Specifically, high cell density induces E-cadherin-mediated recruitment of NF2 and activation of the MST1/2-LATS1/2 cascade, which phosphorylates and retains YAP/TAZ in the cytoplasm, inhibiting their transcriptional activation of ACSL4 and TFR1, ultimately contributing to ferroptosis resistance.^47,260^ Consistently, various post-transcriptional modifications regulate the expression and activity of YAP protein, impacting ferroptosis susceptibility. Cyclin-dependent kinase 7 (CDK7) independently promotes nuclear YAP phosphorylation at the S127 and S397 sites, inducing downstream LDHD protein expression and D-lactate-induced ferroptosis resistance in esophageal squamous cell carcinoma (ESCC).^261^ Glutamine-fructose-6-phosphate transaminase (GFPT1) maintains YAP stability through o-GlcNAcylation, countering Hippo pathway suppression. Inhibition of system xc^-^ impairs this process, reducing ferritin levels, increasing intracellular iron, and enhancing ferroptosis sensitivity.^262^ Furthermore, TAZ can activate NOX2/4 to promote ferroptosis by upregulating angiopoietin-like 4 (ANGPTL4) in ovarian cancers or epithelial membrane protein 1 (EMP1) in renal cancers.^259,263,264^ However, the ferroptosis sensitivity of the Burkitt lymphoma cell lines, which do not express YAP or its homolog TAZ, can still be influenced by cell density, indicating an alternative mechanism and the limited role of YAP/TAZ in cell density-mediated ferroptosis vulnerability.^47^

### Autophagy pathway

Ferroptosis exhibits a dependence on autophagy in various induction mechanisms,^265^ referring to ferritinophagy, lipophagy, mitochondrial autophagy, clockophagy, and chaperone-mediated autophagy (CMA) (Fig. 3h). Ferritinophagy involves autophagic degradation of ferritin, facilitated by NCOA4 binding and subsequent delivery to lysosomes.^266^ Ataxia telangiectasia mutated (ATM) phosphorylates NCOA4 to enhance ferritinophagy, promoting ferroptosis by increasing intracellular labile iron.^267^ Conversely, tripartite motif-containing protein 7 (TRIM7) ubiquitinates and degrades NCOA4, inhibiting ferritinophagy and tumor cell sensitivity to ferroptosis.^268^ Lipophagy targets lipid droplets for lysosomal degradation, providing substrates for lipid peroxidation.^269^ RAB7A, member of the RAS oncogene family, enhances lipophagy-mediated ferroptosis by promoting autophagosome formation.^269,270^ Progesterone receptor membrane component 1 (PGRMC1) enhances ferroptosis susceptibility through silent information regulator 1 (SIRT1) activation-mediated lipophagy.^271^ Mitophagy and clockophagy selectively degrade mitochondria and ARNTL, respectively, promoting ferroptosis by inducing mitochondrial depletion and inhibiting fatty acid uptake and lipid storage.^231,272,273^ CMA, a highly selective autophagy pathway independent of vesicles, relies on chaperone proteins and lysosome-associated membrane protein 2a (LAMP2A) to deliver ferroptosis-related proteins to lysosomes for degradation, regulating ferroptosis in tumor cells.^274^ GPX4, a common substrate protein, undergoes CMA degradation facilitated by heat shock cognate 71 kDa protein (HSC70) and heat shock protein 90 (HSP90), enhancing the sensitivity of tumor cells to ferroptosis.^275,276^ Creatine kinase B (CKB) inhibits CMA-mediated GPX4 degradation by phosphorylating GPX4 and preventing its interaction with HSP70, providing protection against ferroptosis.^276^ However, most studies supporting the autophagy dependence of ferroptosis focus on the late stages of the ferroptosis process. This adds uncertainty to the concept of autophagy dependence in ferroptosis, as, in the late stages of oxidative damage, the mixed forms of cell death involving autophagy may become more common.^277–280^ Therefore, further research is required to clarify the permissive or regulatory role of autophagy in the process of ferroptosis.

### Metabolism pathway

Energy metabolism is responsible for sustaining fundamental biological activities. Mitochondria serves as the primary energy production and acts as the main regulator for ROS stress and antioxidant defense.^281,282^ Glutaminolysis, TCA cycle and electron transport chain (ETC) are crucial for cysteine starvation-induced ferroptosis^160^ (Fig. 3i). Glutaminolysis metabolism fuels the TCA cycle by converting intracellular glutamine to glutamate via glutaminase (GLS), which is further metabolized to alpha-ketoglutarate (αKG) in mitochondria via glutamate dehydrogenase 1 (GLUD1). Glutaminolysis inhibition disrupts cystine deprivation-induced ferroptosis, whereas TCA metabolites downstream of αKG, including succinate, fumarate and malate can restore the role of glutamine in ferroptosis.^160,283,284^ Inhibiting the mitochondrial ETC also attenuates ferroptosis induced by cystine deprivation, as does depletion of mitochondria,^160^ partly due to the less leakage of electrons that produce superoxide and H_2_O_2_, which can then react with ferrous iron to drive Fenton chemistry and lipid peroxidation.^12^ The pentose phosphate pathway (PPP) contributes to ferroptosis by generating NADPH, which is involved in various defense mechanisms against ferroptosis, including GSH reduction,^285^ and the synthesis of thioredoxin and CoQ_10_.^116,286^ Inhibition of PPP-related enzymes impedes erastin-induced ferroptosis.^4^ Furthermore, glucose deprivation-induced energy stress activates AMP-activated protein kinase (AMPK), inhibiting PUFA biosynthesis and conferring ferroptosis resistance via ACC phosphorylation.^287^ The activation of AMPK in response to energy stress could also be regulated by liver kinase B1 (LKB1), which negatively regulates ferroptosis through AMPK/ACC-mediated PUFA inhibition.^287,288^

Lipid, amino acid, and vitamin metabolism also regulate ferroptosis sensitivity. As noted above, lipid droplet degradation, PUFA/MUFA phospholipid activation/synthesis, and PUFA-PL oxidation are essential in ferroptosis.^4,44,141,269–271^ Moreover, high-fat diet downregulates ACSL4 and promotes tumor cell invasiveness and resistance to ferroptosis.^289^ Chronic exposure to 27-hydroxycholesterol enhances GPX4 expression in ER-breast cancer cells, counteracting metabolic stress and leading to ferroptosis resistance.^290^ Adipokine inhibits ferroptosis by suppressing fatty acid oxidation and maintaining lipid levels via HIF2α activation.^291^ Additionally, cysteine starvation and glutamine supplementation induce or promote ferroptosis.^292,293^ Interestingly, prolonged methionine deprivation prevents GSH depletion from ferroptosis, whereas short-term methionine starvation promotes ferroptosis by stimulating CHAC1 transcription.^294^ Tryptophan facilitates cancer cells to escape from ferroptosis through its metabolites serotonin and 3-hydroxyanthranilic acid as radical-trapping antioxidants.^295^ Kynurenine, a product of tryptophan oxidation, also inhibits ferroptosis by scavenging ROS and activating NRF2 activity.^296^ Vitamin E is a well-known inhibitor of ferroptosis, both in vivo and in vitro, due to its powerful antioxidant properties.^191,297,298^ Vitamin K also inhibits ferroptosis by reducing to hydroquinone via FSP1 and vitamin K epoxide reductase complex subunit 1 like 1 (VKORC1L1).^56,299^

## Ferroptosis-mediated crosstalk within the tumor microenvironment (TME)

The TME is a dynamic and complex ecosystem comprising cancer cells, stromal cells, diverse subpopulations of immune cells, the blood and lymphatic vasculature, and various acellular components.^300^ In the TME, bidirectional communication between cancer cells and their microenvironment is critical for tumor growth.^301^ In particular, dying cancer cells communicate with immune cells through the exposure or release of multiple signals during ferroptosis, thus modulating the anti-tumor immune responses. Simultaneously, mediators released by immune cells also have a crucial impact on regulating the susceptibility of cancer cells to ferroptosis. Pharmacologic screening identifies that CD8^+^ T cells exhibited a higher sensitivity to FINs than cancer cells,^302^ suggesting that pro-ferroptotic stimuli could elicit ferroptosis not only in cancer cells but also in tumor-infiltrating immune cells. Correspondingly, the occurrence of ferroptosis in immune cells will affect their survival and immunomodulatory function, ultimately reprograming tumor progression in the TME. Therefore, the versatile and complex roles of ferroptosis in the crosstalk between tumor cells and nonmalignant cells, particularly immune cells, within the TME are discussed below.

### Immunomodulatory role of ferroptotic cancer cells

**Fig. 4:**
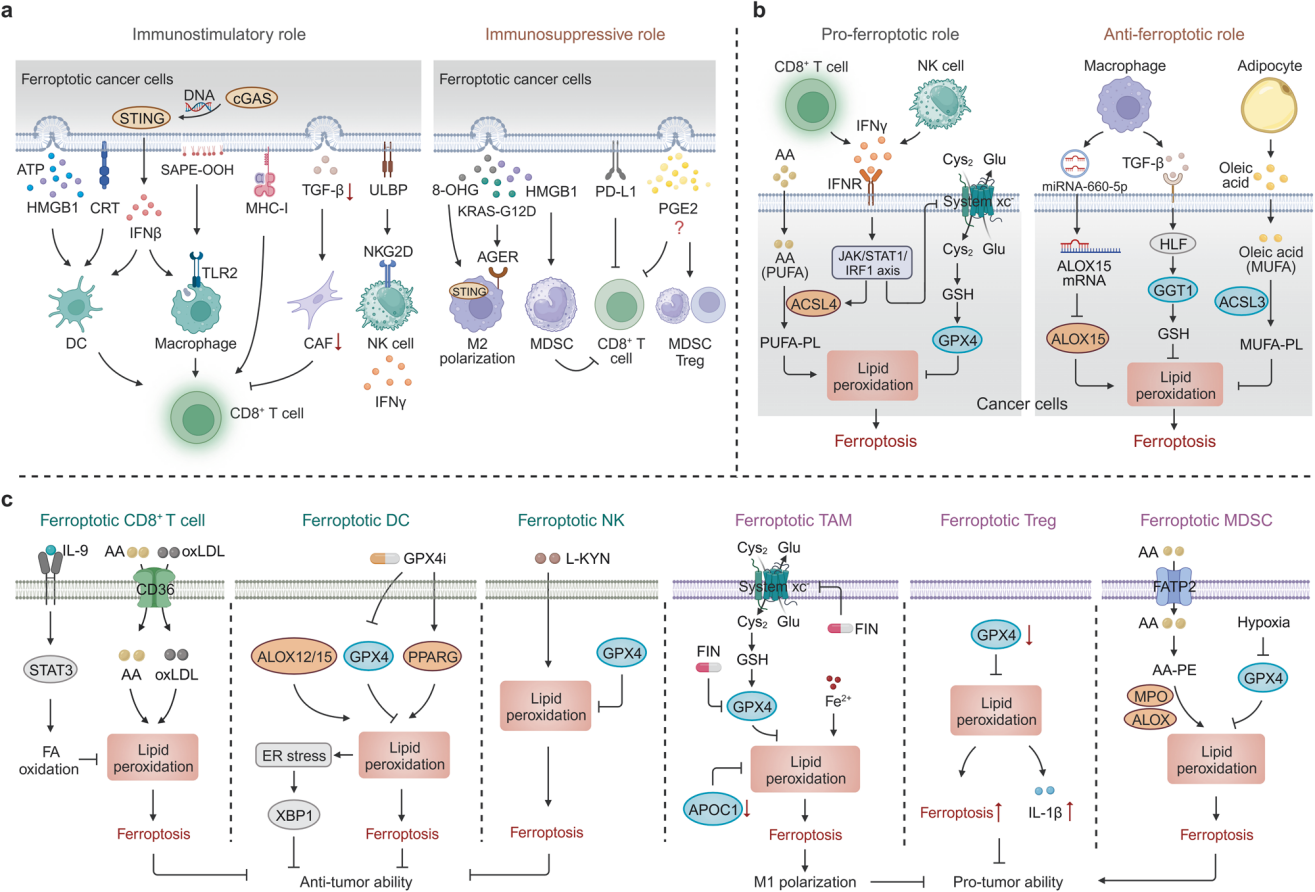
Ferroptosis-mediated crosstalk in the tumor microenvironment (TME). **a** Ferroptotic cancer cells in the TME exhibit dual immunoregulatory effects, encompassing both immunostimulatory and immunosuppressive roles. The emission of various immunomodulatory signals by ferroptotic cancer cells activates different immune responses regulating tumor development. **b** The pro-ferroptotic and anti-ferroptotic impact on cancer cells mediated by immune cells and adipocytes in the TME. **c** The mechanisms and tumor-modulating effects of ferroptotic immune cells in the TME, including CD8^+^ T cells, dendritic cells (DCs), natural killer (NK) cells, tumor-associated macrophages (TAMs), regulatory T cells (Tregs), and myeloid-derived suppressor cells (MDSCs). AA arachidonic acid, AGER advanced glycosylation end product-specific receptor, CAF cancer-associated fibroblast, CRT calreticulin, FATP2 fatty acid transport protein 2, FIN ferroptosis inducer, HMGB1 high-mobility group box 1, IFNγ interferon gamma, 8-OHG 8-hydroxy-2-deoxyguanosine, oxLDL oxidized low-density lipoproteins, STING stimulator of interferon genes, TGF-β, transforming growth factor beta, TLR2 Toll-like receptors 2, ULBP UL16 binding protein. This figure was created with BioRender.com

The emission of immunomodulatory signals by ferroptotic cancer cells, such as damage-associated molecular patterns (DAMPs), MHC class I molecules, cytokines, and lipid metabolites, exerts a significant and multifaceted impact on tumor growth by activating distinct immune responses (Fig. 4a).

### Immunostimulatory activities of ferroptotic cancer cells

Cancer cells undergoing RCD, including ferroptosis, could elicit protective anticancer immunity by emitting a series of endogenous adjuvant signals that are generally referred to as DAMPs.^303^ Under pro-ferroptotic stress, the intracellular DAMPs, including double-stranded DNA and mitochondrial DNA, can activate the cyclic GMP-AMP synthase (cGAS)/stimulator of interferon genes (STING) pathway, leading to the release of interferon β (IFNβ).^304–306^ Subsequently, IFNβ enhances dendritic cell (DC) maturation, macrophage phagocytosis, and the infiltration of cytotoxic CD8^+^ T cells, thereby resulting in tumor regression in preclinical cancer models.^304–306^ Additionally, oxidative stress triggers the upregulation and translocation of the calreticulin on the surface of ferroptotic cancer cells.^306–308^ Calreticulin serves as an ‘eat-me’ signal, promotes DC maturation, and increases the infiltration of cytotoxic CD8^+^ T cells into tumors, thus boosting anti-tumor immune responses.^306–308^ Likewise, oxidized PE, 1-steaoryl-2-15-HpETE-sn-glycero-3-phosphatidylethanolamine (SAPE-OOH), another ‘eat-me’ signal, accumulates on the membranes of ferroptotic cancer cells and could be directly recognized by its counterpart, the macrophage Toll-like receptors 2 (TLR2).^309^ This process promotes macrophage-mediated phagocytosis and elimination of ferroptotic cancer cells, thereby inhibiting tumor growth.^309^ Ferroptotic tumor cells also secrete high-mobility group box 1 (HMGB1) and ATP, the best-characterized DAMPs involved in immunogenic cell death.^306,308,310,311^ Notably, only early (1-3 hours), but not late (24 hours) ferroptotic cells release sufficient ATP and HMGB1 to stimulate DC maturation and elicit a vaccination-like anti-tumor immune response.^310^ Hence, further investigation is required to understand the immunostimulatory properties of these signals at different stages of ferroptotic cancer cells, which may contribute to the advancement of cancer vaccines based on ferroptosis.

In addition to DAMPs, pro-ferroptotic stimulation can upregulate other immunoregulatory molecules on the surface of ferroptotic tumor cells or regulate the secretion of cytokines into the TME, thereby boosting anti-tumor immune responses. For instance, inhibition of alpha 1,3-mannosyltransferase (ALG3) stimulates ferroptosis in cancer cells, which leads to the upregulation of MHC class I molecules on the cell surface,^312^ facilitating the infiltration of cytotoxic CD8^+^ T cells and subsequent tumor reduction.^312^ Furthermore, the interaction between the NK cells activating receptor NKG2D and its ligand UL16 binding protein (ULBP) is implicated in ferroptosis-mediated anti-tumor surveillance.^313^ Mechanistically, pro-ferroptosis nanoparticle promotes the upregulation of ULBP on the tumor cell surface, further activate NK cells with increased IFNγ secretion and lytic degranulation, and thus inhibit tumor growth in vivo.^313^ Cytokines secreted by cancer cells play a significant role in manipulating immune functions and guiding cancer progression.^314^ The cytokine transforming growth factor β (TGFβ) is responsible for cancer-associated fibroblast (CAF) formation and the establishment of an immunosuppressive TME after tumorigenesis.^315,316^ Recently, gastrointestinal cancer cells with anoctamin 1 (ANO1) high expression can release TGFβ through inhibiting ferroptosis to facilitate CAF recruitment and cripple CD8^+^ T cell-mediated anti-tumor immunity.^317^ Inhibition of ANO1 promotes an immune-activated TME with impaired TGFβ secretion, which can be restored by ferroptosis inhibitors in vivo, highlighting the significant role of ferroptosis in regulating cytokine release and orchestrating the TME.^317^ In summary, tumor cells could modulate the exposure or release of DAMPs, immunostimulatory molecules, and cytokines upon stimulation by ferroptosis, ultimately enhancing anti-tumor immunity and leading to tumor suppression.

### Immunosuppressive activities of ferroptotic cancer cells

Intriguingly, the release of DAMPs triggered by cancer cells undergoing ferroptosis is a double-edged sword that not only boosts anti-tumor immune cell function but also enhances tumor-promoting responses of immunosuppressive cells in specific contexts.^318^ Ferroptotic damage induces the release of 8-OHG from pancreatic cells, which is a marker of oxidative DNA damage and serves as a DAMP.^319,320^ The released 8-OHG activates the STING-dependent DNA sensor pathway that enhances the infiltration and M2 polarization of macrophages, facilitating pancreatic carcinogenesis.^319^ In addition, the KRAS oncoprotein with G12D mutation is also released as a DAMP by ferroptotic pancreatic cancer cells and can be engulfed by macrophages via the advanced glycosylation end product-specific receptor (AGER) and promotes fatty acid oxidation driven by signal transducer and activator of transcription 3 (STAT3)-dependent in macrophages.^321^ Activation of the AGER-STAT3 pathway ultimately leads to pro-carcinogenic M2 macrophage polarization, and blocking this pathway or ferroptosis with ferrostatin-1 could inhibit TAM-mediated pancreatic tumor growth.^321^ In a hepatocellular tumorigenic model, GPX4 deletion-induced ferroptosis results in the release of high levels of HMGB1, thereby promoting the recruitment of immunosuppressive MDSCs.^322^ GPX4-deficient liver tumors also increase the expression of programmed cell death ligand 1 (PD-L1). Thus, MDSC infiltration and the concomitant PD-L1 upregulation counteract the cytotoxic CD8^+^ T cell response elicited by ferroptotic liver tumor cells, ultimately leading to no significant tumor suppression.^322^

Ferroptotic cancer cells could also release immunosuppressive lipid mediators that favor immunosuppressive responses. It is well-established that PTGS2, a gene that encodes cyclooxygenase-2 and determines the production of prostaglandin E2 (PGE2), is upregulated during ferroptosis in cancer cells.^43^ PGE2 is an immunosuppressive prostanoid lipid that could impair the anti-tumor activity of conventional type 1 DC (cDC1), NK cells, and effector T cells.^323–325^ Meanwhile, PGE2 can activate immunosuppressive cells such as MDSCs and regulatory T cells (Tregs), contributing to immune escape.^325,326^ Overall, ferroptotic tumor cells could emit multiple immunosuppressive signals, especially DAMPs and lipid metabolites, thereby facilitating tumorigenesis and tumor growth.

### Effect of immune cells on cancer cells ferroptosis

Anti-tumor immune cells exert their functions partially by releasing mediators such as cytokines, which can enhance the susceptibility of tumor cells to ferroptosis **(**Fig. 4b**)**. IFNγ released by cytotoxic CD8^+^ T cells binds to its receptor and activates the Janus kinase (JAK)/signal transducer and activator of transcription 1 (STAT1) pathway in cancer cells, leading to the suppression of transcription and expression of SLC3A2 and SLC7A11, the two subunits of the cystine antiporter system xc.^-^^19,327,328^ Hence, system xc^-^ downregulation dampens the import of cystine and enhances lipid peroxidation, thereby rendering cancer cells vulnerable to ferroptosis triggered by pharmacological manipulations.^19,329^ Subsequent research has shown that CD8^+^ T cell-released IFNγ cooperates with AA to directly cause cancer cell ferroptosis in an ACSL4-dependent manner.^330^ Mechanistically, IFNγ activates the JAK/STAT1/interferon regulatory factor 1 (IRF1) signaling pathway and promotes IRF1 to bind to the IFN-stimulated response elements in the ACSL4 promoter region, ultimately leading to ACSL4 transcriptional upregulation in cancer cells.^330,331^ ACSL4 functions by facilitating the incorporation of PUFAs (including AA) into PLs on the plasm membrane.^46^ Therefore, it is not surprising that IFNγ released by activated cytotoxic CD8^+^ T cells reprograms lipid patterns in the presence of AA via ACSL4, thereby inducing and enhancing ferroptosis in cancer cells and resulting in tumor reduction.^332,333^ These studies suggest that IFNγ and AA induce ferroptosis in cancer cells.^19^ In addition to activated CD8^+^ T cells, NK cells are also significant producers of IFNγ.^334^ A recent study has indicated that chimeric antigen receptor (CAR)-modified NK cells, genetically engineered immune cells, could promote cancer cell ferroptosis by releasing IFNγ. Similar to CD8^+^ T cells, IFNγ produced by CAR NK cells enhances ferroptosis seemingly through downregulating the system xc^-^ subunits (SLC3A2 and SLC7A11) in cancer cells.^335^ Nevertheless, the precise mechanism by which CAR NK cells inhibit their expression requires further investigation. In summary, tumor-infiltrating activated CD8^+^ T cells and CAR-modified NK cells can kill cancer cells by enhancing their vulnerability to ferroptosis through the production and release of IFNγ.

Immunosuppressive TAMs have been reported to hinder the ferroptosis of tumor cells by secreting cytokines and microRNAs into the TME, thereby supporting tumor growth. For instance, TAMs can release the cytokine TGFβ1, which binds to its receptor and promotes SMAD family member 3 (SMAD3) on the promoter region of hepatic leukemia factor (HLF) in TNBC cells.^336^ Increased HLF transcription, in turn, enhances the transcription of gamma-glutamyltransferase 1 (GGT1), an enzyme that increases intracellular cysteine availability for GSH synthesis.^337^ Accordingly, TAM-derived TGFβ1 suppresses ferroptosis by boosting the GGT1/GSH/GPX4 axis in TNBC cells. Intriguingly, in addition to GGT1, HLF also induces interleukin 6 (IL6) transcription, activating the JAK2/STAT3 axis to augment TGFβ1 secretion by TAMs, ultimately constituting a feedforward circuit to promote TNBC tumor growth.^336^ In addition, TAMs package the miRNA-660-5p into exosomes, which are secreted into the TME and internalized by cervical cancer cells to interfere with ALOX15 expression.^338^ Downregulation of ALOX15 is involved in cervical cancer cell resistance to ferroptosis.^338^ Furthermore, the role of cancer-associated adipocytes in fueling cancer has gained increasing attention, and this pro-tumor effect may be associated with ferroptosis resistance.^196,339^ Mammary adipocytes secrete oleic acid, a MUFA, which impairs lipid peroxidation and ferroptosis in TNBC cells through an ACSL3-dependent mechanism.^196^ Taken together, TAMs and cancer-associated adipocytes could secrete cytokines, microRNAs, and lipid metabolites to shield cancer cells from ferroptosis and promote tumor progression.

### Effects of ferroptotic immune cells on cancer cells

Emerging evidence has revealed the occurrence of ferroptosis in immune cells within the TME, in addition to cancer cells. This ferroptotic process affects not only the survival of tumor-infiltrating immune cells but also their immunoregulatory properties, ultimately regulating cancer behavior. Herein, a comprehensive understanding of the role of ferroptotic immune cells, including CD8^+^ T cells, B cells, DCs, NK cells, TAMs, Tregs, and MDSCs, in cancer progression will help develop ferroptosis-targeted immunotherapeutic strategies (Fig. 4c).

CD8^+^ T cells are essential for effective anti-tumor immune responses, and ferroptosis-associated lipid metabolism reprogramming contributes to the impairment of CD8^+^ T cells in the TME.^340^ Fatty acids, particularly AA from the TME, facilitate lipid peroxidation and ferroptosis in tumor-infiltrating CD8^+^ T cells through the fatty acid transporter CD36.^341^ This CD36-mediated ferroptosis hampers cytotoxic cytokine production and anti-tumor function of CD8^+^ T cells, with decreased levels of IFNγ, TNFα, and perforin.^341^ Adoptive transfer of CD8^+^ T cells treated with the ferroptosis inhibitor ferrostatin-1 improves the survival of tumor-bearing mice and reduces tumor burden, suggesting that targeting ferroptosis in CD8^+^ T cells could enhance anti-tumor efficacy in vivo.^341^ In addition to AA, CD36 can enhance the absorption of oxidized low-density lipoproteins (OxLDL) into intratumoral CD8^+^ T cells, which induces lipid peroxidation and dysfunction in CD8^+^ T cells.^342^ Importantly, compared to Tc1 cells, IL-9-secreting CD8^+^ Tc9 cells could activate the IL-9/STAT3/fatty acid oxidation pathway, which protects against tumor- or ROS-induced lipid peroxidation and ferroptosis within the TME.^343^ Consistently, STAT3 inhibitor-treated Tc9 cells exhibit increased lipid peroxidation and compromised anti-tumor ability, whereas ferroptosis inhibitor-treated Tc9 cells display reduced iron levels and lipid peroxidation, as well as stronger anti-tumor ability in vivo.^343^ These findings suggest that inhibiting ferroptosis in CD8^+^ T cells can augment anti-tumor immunity and kill tumor cells better.

B cells exhibit remarkable heterogeneity and play complex roles within the TME.^344^ Up to now, the role of ferroptosis in regulating the homeostasis and immune responses of tumor-infiltrating B cells has not been extensively reported. Emerging evidence indicates that marginal zone B cells and B1 cells, but not follicular B2 cells, are susceptible to GPX4 inhibition-induced ferroptosis due to their high expression of CD36 and consequent fatty acid uptake.^345^ Moreover, ferroptosis has been observed in B cells from both systemic lupus erythematosus patients and mice, suggesting that it may regulate B cell differentiation and plasma cell formation to participate in the pathogenesis of lupus.^346^ These studies highlight the significance of ferroptosis in the survival and function of B cells, but the impact of ferroptosis on tumor-infiltrating B cells and B cell-mediated tumor immunity remains to be further investigated.

DCs are recognized as antigen-presenting cells and powerful initiators of T-cell responses that eliminate tumor cells within the TME.^347^ Recent evidence indicates that pro-ferroptotic regulators can impair the anti-tumor function of tumor-infiltrating DCs. Damaging molecules, including ROS and lipid peroxidation byproduct 4-HNE, the marker of ferroptosis, accumulate in ovarian cancer-associated DCs.^348,349^ This accumulation promotes endoplasmic reticulum stress response and X-box binding protein 1 (XBP1) activation, ultimately impairing the ability of tumor-associated DCs to present antigens and initiate anti-tumor T-cell responses.^348,350^ Intriguingly, GPX4 inhibitor, but not SLC7A11 inhibitor, could trigger ferroptosis in DCs in a PPARG-dependent manner.^351^ Genetic inhibition of PPARG significantly restores the impaired anti-tumor activities of ferroptotic DCs in vivo.^351^ Besides, in an inflammatory model, the enzymatic production of lipid peroxides mediated by ALOX12/15 disrupts the maturation and activation of DCs via NRF2, but the exact role of ALOX12/15-triggered dysfunction of DCs in anti-tumor immunity within the TME requires further clarification.^352^ Collectively, ferroptosis can occur in DCs and cripple their normal anti-tumor function.

Dysfunction of NK cells, a subset of natural cytotoxic lymphocytes, in the TME due to lipid peroxidation-associated oxidative stress favors tumor growth.^353^ L-kynurenine (L-KYN), a tryptophan metabolite in gastric cancer TME, has been reported to trigger lipid peroxidation and ferroptosis in NK cells, thereby facilitating tumor growth in vivo.^354^ Overexpression of GPX4 confers resistance of NK cells to ferroptosis induced by L-KYN within the TME and augments NK cell-mediated tumoricidal effects in vivo.^354^ These findings suggest that TME can render NK cells susceptible to ferroptosis and lead to their dysfunction, highlighting the therapeutic potential of inhibiting NK cell ferroptosis within the TME for cancer therapy.

TAMs exhibit strong plasticity and can differentiate into either immunostimulatory M1 phenotype or immunosuppressive M2 phenotype.^355^ Notably, M1 macrophages display increased resilience against ferroptosis compared to the M2 phenotype despite similar expression levels of GPX4, ACSL4, and LPCAT3 between the two subtypes.^356^ This resistance is attributed to the elevated levels of inducible nitric oxide synthase (iNOS) and NO• in M1 macrophages, which can substitute for GPX4 and inhibit ALOX15-mediated lipid peroxidation and ferroptosis.^356^ GPX4 inhibitor RSL3 effectively induces ferroptosis in M2 macrophages while sparing M1 macrophages.^356^ In addition, pro-ferroptotic stimuli can re-educate TAMs into an anti-tumorigenic M1 phenotype through multiple reprogramming pathways during ferroptosis, thereby inhibiting tumor progression. For instance, inhibition of apolipoprotein C1 (APOC1) or SLC7A11 promotes ferroptosis in TAMs, which is characterized by increased iron content, downregulated anti-ferroptosis mediators (GPX4, NRF2, SLC7A11, and GSH), and significant ferroptosis-associated mitochondrial changes.^357,358^ These pro-ferroptosis modifications by APOC1 and SLC7A11 further increase CD86 expression of the M1 phenotype and decrease the expression of CD206, CD163, and ARG1 of M2 phenotype in TAMs, thus inhibiting pro-tumoral M2 polarization and the development of HCC.^357,358^ Additionally, several pro-ferroptosis nanoparticles, such as iron-based metal-organic frameworks loaded with FINs (RSL3 or dihydroartemisinin), drive multiple signaling pathways to shift TAMs from the M2 to M1 phenotype.^359–361^ Ultimately, the shift from M2 to M1 phenotype provokes strong anti-tumor activities of TAMs with phagocytic killing and metastasis inhibition.^359,360,362^ These studies highlight that targeting ferroptosis in TAMs is promising to eliminate pro-tumorigenic M2 macrophages or reprogram TAMs towards a tumoricidal M1 type, thereby inhibiting tumor progression.

Activated Tregs represent a crucial barrier against autoimmunity as well as anti-tumor immunity.^363^ Gpx4-deficient activated Tregs are susceptible to ferroptosis and exhibit enhanced production of the proinflammatory cytokine IL-1β, leading to a promotion of T helper cell 17 (Th17) responses. This process compromises the immunosuppressive function of Tregs within the TME and limits tumor growth in vivo.^364^ Ferroptosis inhibitor administration indeed restores tumor burden in mice with Treg-specific deletion of GPX4. Collectively, targeting ferroptosis by inhibiting GPX4 in intratumoral Tregs seems to be a promising strategy for reprogramming the TME and treating cancer. However, it is worth mentioning that non-selective deletion of GPX4 in Tregs not only elicits anti-tumor immunity but also detrimental autoimmunity, such as significant inflammation in the colon.^364^ Therefore, future studies should further determine how to selectively target tumor-infiltrating Tregs without affecting Tregs in healthy tissues when inducing ferroptosis to avoid systemic loss of immune tolerance.

MDSCs are pathologically activated immature cells with potent immunosuppressive effects and great heterogeneity.^365^ They can be identified as two subgroups: PMN- and monocytic (M)-MDSCs.^366^ Tumor-infiltrating PMN-MDSCs, but not M-MDSCs, are vulnerable to ferroptosis or even experience spontaneous ferroptosis within the TME.^23^ Increased AA uptake through the fatty acid transport protein 2 (FATP2) and hypoxia-mediated downregulation of GPX4 both contribute to this susceptibility to ferroptosis.^23^ Although ferroptosis reduces the number of PMN-MDSCs, the increased release of immunosuppressive molecules, such as PGE2 and oxidized lipids, from ferroptotic PMN-MDSCs promotes tumor growth by restricting anti-tumor T cells and supporting the suppressive activity of TAMs.^23,367^ These findings suggest ferroptosis induction could decrease the viability of MDSCs, but the complex immunoregulatory nature of ferroptosis in MDSCs, as well as TME, must be considered in further research.

## Therapeutic strategies of ferroptosis in cancer

As noted above, ferroptosis is tightly interwoven with cell metabolic and oxidative burdens, suggesting the possibility that cancer cells may have higher predispositions to FINs for their overall more active metabolism, higher ROS levels and iron requirements.^11,368,369^ Intriguingly, mesenchymal and dedifferentiated cancer cells, which are usually resistant to apoptosis and traditional therapies, are exquisitely vulnerable to ferroptosis.^182,247,370^ Therefore, FINs hold promise in cancer treatment.^371^ However, due to the immunosuppressive regulatory role and the tissue-damaging ability of ferroptosis, ferroptosis inhibition also proves to be an effective strategy to prevent tumor initiation, inhibit tumor progression, improve tissue damage-mediated cachexia in advanced tumors, and alleviate the side effects of traditional therapies. For these reasons, a comprehensive understanding of the current applications of both ferroptosis induction and inhibition in cancer will pave the way for their clinical implementation.

### Ferroptosis induction

**Fig. 5:**
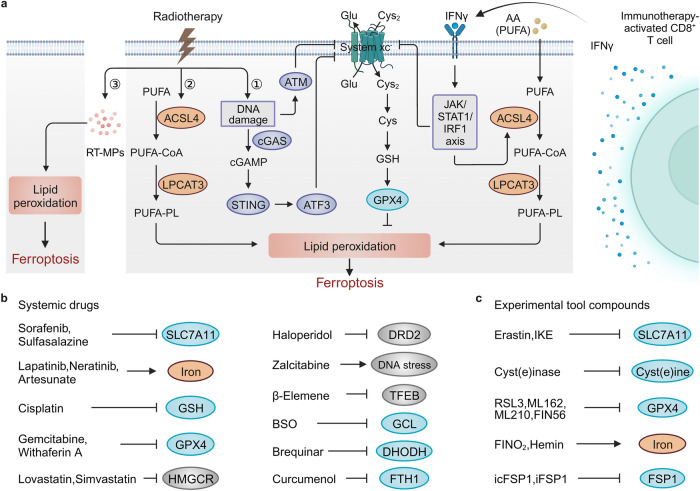
Ferroptosis induction for cancer therapy. **a** Radiotherapy induces ferroptosis to suppress tumor through the following three mechanisms: ① Radiotherapy-induced DNA damage activates the ATM and cGAS/STING/ATF3 axis, leading to SLC7A11 inhibition and subsequent triggering of ferroptosis.② Radiotherapy upregulates the expression of ACSL4, facilitating the PUFA-PLs formation and inducing ferroptosis. ③ RT-MPs induce ferroptosis in neighboring unirradiated cells relying on the bystander effect. After immunotherapy treatment, activated CD8^+^ T cells release IFNγ, sensitizing tumor cells to ferroptosis by inhibiting SLC7A11, and promoting ACSL4-mediated PUFA-PLs formation, ultimately triggering ferroptosis. Immunotherapy and radiotherapy synergistically inhibit tumors by suppressing SLC7A11. **b**, **c** Major systemic drugs and experimental tool compounds for effective treatment of tumors through ferroptosis induction. ATF3 activation transcription factor 3, cGAS cyclic GMP-AMP synthase, cGAMP cyclic 2’,3’-GMP-AMP, DHA dihydroartemisinin, DHODH dihydroorotate dehydrogenase, FSP1 ferroptosis suppressor protein 1, FTH1 ferritin heavy chain 1, GCL glutamate-cysteine ligase, IFNγ interferon gamma, IKE imidazole ketone erastin, RT-MPs irradiated tumor cell-derived microparticles, STING stimulator of interferon genes. This figure was created with BioRender.com

In addition to immunotherapy and radiotherapy,^19,372^ a diverse range of systemic drugs, including but not limited to targeted therapy,^214^ chemotherapy,^138,373^ lipid-lowering drugs,^374,375^ and anti-inflammatory drugs,^376,377^ have been identified as FINs and possess tumor-suppressive abilities.^60^ **(**Table 1**)**. Here, we will describe in detail FINs that have previously entered clinical trials and briefly discuss the main tool compounds used to induce ferroptosis (Fig. 5) (Table 2).

**Table 1:** Clinical trial drugs inducing ferroptosis for antitumor treatment

Drugs	Target	Cancer type	Indication	NCT	Phase	References (PMID)
Sorafenib	SLC7A11	HCC GC CCRC	HCC AML Neuroblastoma Lung cancer	NCT03794440 NCT03247088 NCT02559778 NCT00064350	Marketed	26403645 36473315 31899616 37713596
Sulfasalazine	SLC7A11	Prostate cancer Lymphoma Lung cancer CRC HNC PDAC OCCC	GBM GBM Breast cancer Solid tumor	NCT04205357 NCT01577966 NCT03847311 NCT01198145	Marketed as an anti-inflammatory agent, in oncology phase I trials	11587223 31949285 37190291 37132587 27477897 28130223 37511540
Lapatinib	Iron	Breast cancer	Breast cancer Breast cancer Breast cancer	NCT03085368 NCT00356811 NCT00667251	Marketed	27441659
Neratinib	Iron	Breast cancer	Breast cancer CRC CRC	NCT04366713 NCT03377387 NCT03457896	Marketed	37596261
Artesunate	Iron	NHL HCC	Breast cancer CRC	NCT00764036 NCT03093129	Marketed as an antimalarial drug, in oncology phase II trials	37326033 32699265
Cisplatin	GSH	GC HNC NSCLC	NSCLC Bladder cancer Cervical cancer Pancreas cancer	NCT01656551 NCT04574960 NCT01561586 NCT03649321	Marketed	27477897 28012440 35534546 35784745
Gemcitabine	GPX4	PDAC LUAD	Pancreatic cancer BTC Solid Tumor	NCT06015659 NCT05357196 NCT05147272	Marketed	36225575 28130223
Withaferin A	GPX4	Neuroblastoma HCC	Ovarian cancer Advanced cancer Osteosarcoma	NCT05610735 NCT04092647 NCT00689195	Phase II	29939160 36707233
Lovastatin	HMGCR	NSCLC	Prostate cancer Ovarian Cancer	NCT00580970 NCT00585052	Marketed as lipid-lowering agents, in oncology	35943796
Simvastatin	HMGCR	TNBC	Multiple myeloma	NCT00281476	phase II trials	34627266
Haloperidol	DRD2	GBM	Advanced cancer	NCT04833023 NCT03743649 NCT00124930	Marked for the treatment of psychiatric disorders, phase IV for the treatment of cancer	37249604
Zalcitabine	DNA stress	Pancreatic cancer	AIDS-related Kaposi sarcoma	NCT00000954	Marketed for the treatment of HIV, phase I for the treatment of cancer	32186434
β-Elemene	TFEB	NSCLC	NSCLC GBM	NCT03123484 NCT02629757	Marketed	37689240
BSO	GCL	TNBC	Neuroblastoma Neuroblastoma	NCT00005835 NCT00002730	Phase I	37563614 37256771
Brequinar	DHODH	Cervical cancer Colon cancer Fibrosarcoma Lung cancer	AML	NCT03760666	Phase II	33981038 37291265 36672495
Curcumenol	FTH1	Lung cancer	Cancer	NCT00475683	Phase III	35224289

*NCT* national clinical trial, *N/A* not applicable, *BSO* buthionine sulfoxide amine, *GPX4* glutathione peroxidase 4, *GSH* glutathione, *SLC7A11* solute carrier family 7 member 11, *HMGCR* 3-hydroxy-3-methylglutaryl-coenzyme A reductase, *DRD2* dopamine D2 receptor, *TFEB* transcription factor EB, *GCL* glutamate-cysteine ligase, *DHODH* dihydroorotate dehydrogenase, *FTH1* ferritin heavy chain 1, *HCC* hepatocellular carcinoma, *GC* gastric cancer, *CCRC* clear cell renal cell carcinoma, *HNC* head and neck cancer, *NSCLC* non-small-cell lung cancer, *PDAC* pancreatic ductal adenocarcinoma, *LUAD* lung adenocarcinoma, *CRC* colorectal cancer, *OCCC* ovarian clear cell carcinoma, *TNBC* triple-negative breast cancer, *NHL* non‑Hodgkin lymphoma, *AIDs* acquired immunodeficiency syndrome, *BTC* biliary tract cancer, *AML* acute myeloid leukemia, *GBM* glioblastoma

**Table 2:** Classical pharmacological agents inducing ferroptosis for antitumor treatment

Compounds	Target	Mechanism	Cancer type	References (PMID)
Erastin	SLC7A11	GSH depletion by inhibiting SLC7A11 activity	HCC Melanoma NSCLC LUAD Ovarian cancer Anaplastic thyroid cancer	31974380 37277863 31897145 33882617 31800616 36442849 36895980
IKE	SLC7A11	GSH depletion by inhibiting SLC7A11 activity	DLBCL Sarcoma GBM NSCLC	24439385 30799221 31899616 37752118 37957645
Cyst(e)inase	Cyst(e)ine	GSH depletion by degrading cysteine and cystine	Prostate cancer Breast cancer Lung cancer Pancreatic tumors Ovarian cancer	27869804 29168506 32241947 31043744
RSL3	GPX4	GPX4 inactivation	Fibrosarcoma TNBC HCC Lung cancer Anaplastic thyroid cancer	24439385 30545638 36257316 37596261 31556117 36895980
M162	GPX4	GPX4 inactivation	TNBC Anaplastic thyroid cancer	36257316 36895980
ML210	GPX4	GPX4 inactivation	HNC TNBC NSCLC Anaplastic thyroid cancer	33741422 34623753 37655031 36895980
FIN56	GPX4	GPX4 degradation	GBM Lung cancer	34659551 31949285
FINO_2_	Iron and GPX4	Iron oxidation and GPX4 inactivation	Fibrosarcoma	29610484
Hemin	Iron	Iron loading	Lung cancer	36228518
icFSP1	FSP1	Phase separation of FSP1	Melanoma	37380771
iFSP1	FSP1	FSP1 inhibition	TNBC NSCLC HCC	37432874 36893885

*IKE* imidazole ketone erastin, *SLC7A11* solute carrier family 7 member 11, *GPX4* glutathione peroxidase 4, *FSP1* ferroptosis suppressor protein 1, *GSH* glutathione, *HCC* hepatocellular carcinoma, *NSCLC* non-small-cell lung cancer, *LUAD* lung adenocarcinoma, *DLBCL* diffuse large B-cell lymphoma, *TNBC* triple-negative breast cancer, *GBM* glioblastoma, *HNC* head and neck cancer

### Immunotherapy

Immunotherapy with immune checkpoint inhibitors (ICIs) is a promising approach that targets explicitly dysfunctional immune systems and mainly activates CD8^+^ T cells to eradicate tumor cells effectively.^378^ The advent of ICIs, specifically anti-CTLA4 and anti-PD-1/PD-L1 antibodies, have brought about a paradigm shift in cancer therapy and represent a significant breakthrough in oncology.^379^ As described above, on one hand, IFNγ derived from CD8^+^ T cells activates the JAK/STAT1 pathway to downregulate SLC7A11 and SLC3A2, thereby sensitizing tumor cells to ferroptosis^19,380^; on the other hand, IFNγ can transcriptionally stimulate ACSL4 expression to promote the integration of TME-associated AA into PLs by STAT1/IRF1 signaling, ultimately inducing ferroptosis in tumor cells.^330^ Therefore, ferroptosis induction contributes to the anti-tumor effects of CD8^+^ T cells, and immunotherapy can promote cancer cell ferroptosis in vivo. As expected, inhibiting ferroptosis by liproxstatin-1 diminishes the effectiveness of ICIs in controlling tumor growth.^19^ Additionally, the resistance of tumor cells to ferroptosis is associated with unresponsiveness to ICIs. Restoring their sensitivity to ferroptosis could enhance immunotherapy efficacy. TYRO3^high^ tumors, which are resistant to ICIs, can be re-sensitized to anti-PD1 therapy by restoring ferroptosis via inhibiting TYRO3-mediated AKT/NRF2 pathway.^381^

Owing to the immunomodulatory effect of ferroptotic cells and the involvement of ferroptosis in ICIs anti-tumor effect, ferroptosis induction holds promise as an anti-tumor strategy to enhance the efficacy of ICIs. A growing body of evidence has demonstrated that combining ICIs and ferroptosis-inducing agents synergistically inhibits tumor growth in vitro and vivo.^19,309,382^ For example, the combined treatment of GPX4 inhibitors and anti-PD-1 blockade significantly suppressed tumor growth and induced a pronounced immune response with increased proportions of activated CD8^+^ T cells in TNBC tumor-bearing immunocompetent mice.^382^ IL-1β sustains Fe-S cluster maintenance to repress iron accumulation and ferroptosis. The combination of IL-1β blockade and anti-PD-1 antibody leads to enhanced tumor inhibition compared to monotherapy, but this effect could be reversed by liproxstatin-1, indicating the involvement of ferroptosis.^383^ Moreover, we also found that bromodomain containing 4 (BRD4) is upregulated in ICB-resistant melanoma patients, and inhibiting BRD4/AKR1C2 axis by bromodomain and extra-terminal motif (BET) inhibitors has been shown to enhance the susceptibility of melanoma to ferroptosis and immunotherapy.^384^ Nevertheless, the intricate impact of ferroptosis on the TME limits the applicability of this combination strategy due to immunosuppressive activities triggered by ferroptosis. For example, although GPX4 inhibition-induced ferroptosis in HCC cells increased CD8^+^ T cell infiltration, this effect was counteracted by PD-L1 upregulation on tumor cells.^322^ Synchronously, ferroptosis triggered immunosuppressive MDSC infiltration through increased release of HMGB1 from hepatocytes. The triple combination of pharmacological FINs, checkpoint blockade, and MDSC suppression effectively inhibits primary liver tumors and liver metastasis.^322^ Hence, the specific components of multidrug combination therapy based on ferroptosis-inducing agents and immunotherapy could be customized to counteract the immunosuppression triggered by ferroptosis, so as to evoke robust anti-tumor immune responses and enhance the efficacy of anti-tumor treatment.

### Radiation therapy

Radiotherapy, a widely employed cancer treatment modality, involves precisely administering ionizing radiation (IR) to target and eliminate tumor cells selectively.^385,386^ Radiotherapy directly induces diverse forms of DNA damage and is capable of inducing ferroptosis to inhibit tumor growth.^327,387^ The mechanisms through which radiotherapy induces ferroptosis are multifaceted.^18^ Firstly, radiotherapy-induced DNA double-strand breaks (DSBs) downregulate SLC7A11expression in an ATM-dependent manner, resulting in reduced cystine uptake, and enhanced ferroptosis.^327^ Moreover, radiotherapy-induced DNA damage also can activate the cGAS/STING pathway to trigger tumor ferroptosis via activating transcription factor 3 (ATF3) /SLC7A11/GPX4 axis.^304^ Secondly, radiotherapy induces the expression of ACSL4 to promote the integration of PUFAs into PLs, resulting in the formation of PUFA-PLs, ultimately resulting in ferroptosis.^372^ Thirdly, irradiated tumor cell-derived microparticles (RT-MPs) induce a bystander effect via inducing ferroptosis, which causes the generation of oxidative stress and DNA damage in neighboring unirradiated cells.^388^ The mechanism by which RT-MPs induce ferroptosis is not yet fully understood, but the colocalization of RT-MPs membranes with lysosomes and mitochondria provides a direction for understanding its potential mechanisms.^388^

The occurrence of radioresistance, which leads to the failure of radiotherapy, is undeniably linked to metastasis, cancer recurrence, and unfavourable prognosis.^389^ Ferroptosis has also been implicated in radioresistance. IR can induce SLC7A11 and GPX4 upregulated as an adaptive response to safeguard cells against ferroptosis and contribute to radioresistance,^372,390,391^ suggesting that inhibiting SLC7A11 or GPX4 sensitizes radioresistant cancers to IR. As expected, the combination of class I FINs that inhibit SLC7A11 or class II or III FINs that inhibit or deplete GPX4 with IR demonstrated synergistic effects in inducing lipid peroxidation and ferroptosis.^372^ Moreover, suppressors of cytokine signaling 2 (SOCS2) were screened out as a potential biomarker predicting radiosensitivity of HCC. Mechanistically, SOCS2 transfers the attached ubiquitin to SLC7A11 and promotes K48-linked polyubiquitination degradation of SLC7A11.^391^ Conversely, stanniocalcin 2 (STC2) activate PRMT5, leading to upregulation of SLC7A11 and resistance to ferroptosis, thus playing a role in ESCC radioresistance.^390^ Other mechanisms that inhibit ferroptosis also contribute to radioresistance. For example, KEAP1 mutant lung cancers, which are refractory to most therapies, including radiotherapy, exhibit the upregulation of the NRF2/FSP1 anti-ferroptosis axis, resulting in resistance to ferroptosis and radiotherapy.^165^ Targeting FSP1 confers vulnerability to ferroptosis and enhances radiosensitivity in KEAP1 mutant lung cancers.^165^ IR-induced downregulation of copper metabolism MURR1 domain 10 (COMMD10) contributes to the radioresistance because COMMD10 inhibition represses ferroptosis through reducing iron concentration and facilitating HIF1α/SLC7A11 axis.^392^ These findings suggested that ferroptosis inhibition contributes to radioresistance, and combining FINs and IR synergistically induces ferroptosis.

### Systemic drugs

Sorafenib, the first tyrosine kinase inhibitor authorized for the treatment of patients with unresectable HCC, advanced renal cell carcinoma, and differentiated thyroid cancer,^210^ has been shown to trigger ferroptosis by inhibiting system xc- and increasing intracellular iron levels.^393–395^ Therefore, increased SLC7A11 expression and the inhibition of ferritin autophagy contribute to resistance against sorafenib.^234,396,397^ For example, YAP/TAZ maintains the protein stability, nuclear localization, and transcriptional activity of ATF4, synergistically encouraging the expression of SLC7A11 and resulting in resistance against sorafenib-induced ferroptosis.^397^ Activation of ATF2 can inhibit protein degradation of SLC7A11 and mediate resistance to sorafenib-induced ferroptosis in gastric cancer.^396^ Moreover, CISD2 dissociates Beclin-1 from the PI3K-III complex and therefore leads to the inhibition of autophagy and resistance against sorafenib-induced ferroptosis.^398^ Depletion of PTBP1 leads to resistance against sorafenib-induced ferroptosis through disrupting NCOA4 translation and avoiding ferritin autophagy.^399^ However, sorafenib-induced ferroptosis is context-dependent, because sorafenib fails to trigger ferroptosis in various tumor cell lines,^400^ and whether these cells have acquired resistance against sorafenib-triggered ferroptosis needs to be clarified.

Lapatinib and neratinib are both tyrosine kinase inhibitors approved for the treatment of breast cancer. In lapatinib-resistant NSCLC cells, the activation of mTORC1 leads to the upregulation of GPX4 expression and inhibits lapatinib-induced ferroptosis. Therefore, inhibition of GPX4 or mTOR can overcome lapatinib resistance and facilitate lapatinib-induced ferroptosis.^401^ Moreover, the combination of lapatinib and siramesine, a lysosomal destabilizing lysosomotropic drug, synergistically induces ferroptosis through regulating iron homeostasis.^402,403^ Neratinib facilitates ferroptosis and suppresses brain metastasis in human epidermal growth factor receptor 2 (HER2)-positive breast cancer as a neoadjuvant therapy through the elevation of intracellular iron levels.^404^ Moreover, neratinib inhibits acute myeloid leukemia cell proliferation by activating autophagy-dependent ferroptosis.^405^ A recent study revealed that neratinib effectively tackled resistance to RSL3 in non-HER2 amplified luminal breast cancer, and combination treatment with RSL3 and neratinib enhances ferroptosis by increasing mitochondrial iron-dependent ROS production and lipid peroxidation.^406^

Cisplatin is a platinum-based chemotherapeutic agent approved by the Food and Drug Administration (FDA) for oncology use in 1978.^407^ Cisplatin-based regimens remain the mainstay of treatment for a wide range of solid tumors.^408^ The anti-tumor mechanism of cisplatin is mainly mediated by the production of nuclear DNA adducts, which ultimately leads to apoptosis.^409^ However, recent studies have shown that cisplatin can also induce ferroptosis by depleting GSH and inactivating GPX4, providing an alternative mechanism for inhibiting tumor growth.^373^ Consistently, NRF2/SLC7A11 signaling pathway is activated in cisplatin-resistant cancer cells, and inhibiting this pathway could trigger ferroptosis and overcome cisplatin resistance.^410–413^ Combining cisplatin with FINs also may be a better strategy to improve the therapeutic effect of cisplatin.^414,415^ Notably, cisplatin-mediated tumor cell ferroptosis can promote the anti-tumor efficacy of ICI therapy by reprogramming TME characterized by the N1 neutrophil polarization and increased T-cell infiltration and Th1 differentiation in NSCLC.^416^

Gemcitabine (GEM) undergoes a complex intracellular conversion into gemcitabine diphosphate and triphosphate nucleotides, which induce DNA chain termination and interfere with DNA synthesis, conferring potent anti-tumor activity across a wide spectrum of tumors.^417^ The anticancer activity of GEM is associated with the Hsp70 member 5 (HSPA5)/GPX4 pathway-mediated ferroptosis induction.^138,254^ Epigallocatechine gallate or sulfasalazine enhances the sensitivity of gemcitabine in PDAC by inhibiting the HSPA5/GPX4 pathway, thereby disinhibiting ferroptosis.^138^ Consistently, the combination of GEM and IKE shows a synergistic antiproliferative effect on LUAD.^418^ Moreover, PDAC-associated fibroblasts can secrete exosome-derived miR-3173-5p, which inhibits ferroptosis and promotes gemcitabine resistance by targeting ACSL4.^419^

Sulfasalazine (SAS), a clinical anti-inflammatory drug used in rheumatoid arthritis,^420^ induces ferroptosis by inhibiting cystine/glutamate antiporter SLC7A11.^376,377^ Similar to classical system xc^-^ inhibitors such as erastin/IKE, SAS effectively induces ferroptotic cell death in chemotherapy-resistant cells and improves chemotherapy response.^410^ SAS also enhances the therapeutic effectiveness of front-line therapies, such as anthracycline daunorubicin, in acute myeloid leukemia,^421^ as well as paclitaxel in ovarian clear cell carcinoma.^422^ Moreover, SAS, as a radiosensitizer, enhances the therapeutic efficacy of radiotherapy by promoting ferroptosis.^327,372,423^ Notably, a specially designed injectable hydrogel drug delivery system loaded with SAS demonstrates remarkable therapeutic efficacy in combating peritoneal dissemination and malignant ascites in advanced HCC, which is resistant to systemic therapies,^424,425^ particularly when combined with anti-PD-1 immunotherapy.^426^

Statins, a class of clinical drugs aimed at reducing blood cholesterol levels,^427^ including fluvastatin,^428^ atorvastatin,^429^ pravastatin,^430^ lovastatin,^431^ and simvastatin,^375^ are considered attractive FINs in daily practice due to their favorable safety profile.^430^ Statins induce ferroptosis by inhibiting the GSH/GPX4 and FSP1/CoQ_10_/NAD(P)H axes via the mevalonate pathway.^247,375^ Simvastatin inhibits the expression of 3-hydroxy-3-methyl-glutaryl-coenzyme A reductase (HMGCR) to downregulate the mevalonate pathway and GPX4, thereby inducing cancer cell ferroptosis.^375^ Lovastatin induces ferroptosis and converts the immuno-cold phenotype to an inflammatory phenotype in NSCLC by downregulating PD-L1 expression in lung cancer cells, making the tumors more responsive to immunotherapy.^431^

Artemisinin, derived from the Chinese herb *Artemisia annua*,^432^ has been found to have anti-tumor effects in various types of tumors through ferroptosis.^433^ Mechanistically, artemisinin promotes ferritinophagy and increases intracellular free iron levels, and finally leads to ferroptosis and tumor inhibition.^434,435^ Artesunate, as an artemisinin derivative, has demonstrated efficacy in suppressing sunitinib-resistant renal cell carcinoma cells through ferroptosis and cell cycle arrest.^435^ Expectedly, artesunate synergistizes with sorafenib to induce ferroptosis in HCC and non-Hodgkin lymphoma cells.^436,437^ Consistently, dihydroartemisinin, the metabolite of artemisinin,^438^ inhibits lung cancer cells by suppressing the PRIM2/SLC7A11 axis,^439^ and enhances the cytotoxicity of gefitinib in LUAD cells,^440^ sorafenib in HCC,^441^ and cisplatin in PDAC.^442^

Haloperidol, a specific antagonist of dopamine receptor D2 (DRD2) extensively used for the treatment of psychiatric disorders,^443^ exhibits inhibitory effects in cancers by inducing ferroptosis.^444,445^ However, the precise mechanism by which it induces ferroptosis remains unclear, and there may be a potential association with autophagy.^446^ Haloperidol demonstrates synergistic activity with temozolomide in the growth inhibition of glioblastoma multiforme (GBM) through enhancing temozolomide-induced autophagy-mediated ferroptosis by inhibiting DRD2.^444^ Haloperidol can also sensitize the erastin- and sorafenib-induced ferroptosis in HCC by inhibiting the sigma 1 receptor (S1R).^447^

Zalcitabine, also known as 2’, 3’-dideoxycytidine, is used for the treatment of patients infected with the human immunodeficiency virus (HIV) by targeting mitochondrial DNA polymerase gamma (POLG).^448^ Zalcitabine induces autophagy-mediated ferroptosis in pancreatic cancer cells by activating mitochondrial DNA stress and the STING1/TMEM173-dependent DNA sensing pathway.^449^

β-Elemene (β-ELE), derived from *Curcuma wenyujin*, is widely used to treat NSCLC in clinical settings in China.^450^ β-ELE binds to transcription factor (TFEB), which is the key regulator of lysosome biogenesis, and notably activates TFEB-mediated lysosome degradation of GPX4, thus inducing NSCLC ferroptosis and resulting tumor suppression.^451^

Withaferin A (WA) is a bioactive compound derived from the ashwagandha plant, *Withania somnifera*.^452^ WA eradicates high-risk neuroblastoma tumors and suppresses relapse rates by inducing ferroptosis via GPX4 targeting and inactivation.^453^ WA also attenuated sorafenib resistance and metastatic potential by KEAP1/NRF2-associated EMT and ferroptosis.^454^ Moreover, a triple combination of withaferin A, the CXCR2 inhibitor and anti-PD-1 immunotherapy greatly improves the survival of wild-type mice with liver tumors and reduces liver metastasis of colorectal cancer. This effect may be attributed to the triggering of GPX4-associated ferroptotic hepatocyte death, leading to an adaptive immune response characterized by the activation of CD8^+^ T cells, upregulation of PD-L1 on tumor cells, and infiltration of immunosuppressive MDSCs.^322^

Buthionine sulfoxide amine (BSO) activates ferroptosis by targeting GCL for de novo GSH elimination.^455^ BSO induces ferroptotic cell death in lung cancer and HCC.^192,456^ BSO enhances the therapeutic effect of traditional chemotherapy regimens^455^ and radiotherapy in TNBC by promoting ferroptosis.^457^

Brequinar, a DHODH inhibitor, serves as a potential agent to treat GPX4^low^ cancers by triggering ferroptosis,^134^ whereas combined administration of brequinar and sulfasalazine synergistically suppresses GPX4^high^ tumors growth.^134^ Moreover, AMPK activation enhances the assembly of pyrimidinosomes, rendering cancer cells more reliant on DHODH-mediated ferroptosis defense to counteract AMPK-associated stresses.^458^ The combination of brequinar and AMPK activators exhibit synergistic efficacy in tumor suppression through ferroptosis.^458^

Curcumenol, an effective compound found in Wenyujin, has been discovered to inhibit the growth of lung cancer tumors by inducing ferroptosis through lncRNA H19/miR-19b-3p/FTH1 axis.^459^

### Others

In addition to FINs that have previously entered clinical trials, a wide range of experimental tool compounds have been employed in preclinical studies of ferroptosis (Table 2). These compounds could be classified into four classes.^12,460–462^ Classical I FINs are identified through the depletion of GSH to trigger ferroptosis, such as erastin, its derivatives imidazole ketone erastin (IKE),^460,463,464^ and cyst(e)inase, which depletes GSH by degrading cysteine and cystine.^465^ Classical II FINs directly inhibit GPX4 to induce ferroptosis, such as RSL3, ML162 and ML210.^43,269,382^ Classical III FINs deplete the GPX4 protein and CoQ_10_ to induce ferroptosis, such as FIN56.^45,466–469^ Class IV FINs induce ferroptosis by augmenting the LIP, such as FINO_2_.^136^ These tool compounds have made significant contributions to the understanding of the mechanism of ferroptosis due to their specificity towards ferroptosis.

Moreover, the drug delivery system has gained increasing interest in tumor treatment due to its effective delivery and precise control of drug release. Nowadays, three delivery systems, including nanoparticles, hydrogels, and liposomes, have been utilized to improve the efficiency and selectivity of FINs in targeting tumors while minimizing toxicity to normal organs. For example, the acidity-activatable dynamic nanoparticles BNP@R were developed to specifically deliver RSL3 to tumors and enable acid-activatable photodynamic therapy, thereby promoting RSL3-induced ferroptosis and ultimately inhibiting tumor growth.^470^ The tumor-suppressing effect of RSL3 in vivo was also potentiated when delivered in an injectable alginate hydrogel RTFG@SA.^471^ Brequinar-loaded mitochondrial-targeted liposomes BQR@MLipo were employed to enhance brequinar-mediated mitochondrial ferroptosis, effectively inhibiting bladder cancer growth.^306^ The continuous advancements in drug delivery systems targeting ferroptosis contribute to the clinical applications of FINs in cancer treatment.

### Ferroptosis inhibition

The heterogeneity of tumors and the immune microenvironment complicates the role of ferroptosis in tumor suppression. Under some conditions, ferroptosis is even conducive to tumor initiation and progression: 1) The inflammation resulting from ferroptosis-induced tissue damage contributes the onset of necroinflammation-driven tumors.^21,148^; 2) The vulnerability of immune cells to ferroptosis compromises their anti-tumor function or enhances their pro-tumor effect, leading to tumor growth.^23,354,449^; 3) The immunosuppressive activities of ferroptotic cancer cells promotes the tumor progression.^321^ Moreover, it is worth noting that ferroptosis inhibition could also be considered an effective means to suppress the side effects mediated by traditional therapy-induced ferroptosis, due to the tissue-damaging ability of ferroptosis. Thus, ferroptosis inhibition seems to be a potential context-dependent strategy for cancer treatment.

Inhibition of ferroptosis is advantageous in suppressing the initiation of necroinflammation-driven tumors. Liver-related diseases, including steatohepatitis, can trigger the initiation of hepatocyte stress, excessive cell death, subsequent necroinflammation, and compensatory proliferation, which are considered to be the aetiologies of hepatocellular carcinogenesis.^472,473^ A recent study has revealed that ferroptosis stands out as the most pertinent form of hepatocyte death, leading to HCC-promoting necroinflammation and compensatory proliferation.^21^ By contrast, the activation transcription factor 4 (ATF4) attenuates the progression from steatohepatitis to HCC by upregulating SLC7A11 to block stress-related ferroptosis, thereby blunting HCC onset. Moreover, high-iron diets or depletion of Gpx4-induced ferroptosis promotes pancreatitis and pancreatic tumorigenesis. Inhibiting ferroptosis through the administration of liproxstatin-1 reduces the formation of spontaneous pancreatic cancer and reverses the promotion of PDAC development by high-iron diets or GPX4 depletion.^319^ The initiation of ferroptosis-driven pancreatic cancer may be associated with macrophage infiltration and activation, which is mediated by the release of 8-OHG caused by ferroptotic damage and subsequent activation of the STING-dependent DNA sensor pathway in macrophages.^319^

Ferroptosis inhibition rescues immune cells from undergoing ferroptosis and affects their immune regulatory ability, thereby impeding tumor development. CD36-mediated ferroptosis hampers the effector function of intratumoral CD8^+^ T cells and diminishes their ability to combat tumors. Consequently, genetic deletion of CD36 or inhibition of ferroptosis with ferrostatin-1 in CD8^+^ T cells can effectively restore their anti-tumor effects.^449^ NK cells also undergo ferroptosis induced by various triggers in the TME, including L-KYN released by tumor cells and CAF-derived follistatin-like protein 1 (FSTL1) and iron.^354,474^ Overexpression of GPX4 in NK cells effectively inhibits ferroptosis and prevents their reduction within the TME, thereby suppressing tumor growth.^354^ Consistently, the combination of FSTL1-neutralizing antibody and deferoxamine significantly inhibits NK cell ferroptosis, enhancing the cytotoxicity of NK cells against tumor cells.^474^ In addition, tumor-associated PMN-MDSCs undergo ferroptosis spontaneously.^23^ Intriguingly, although this ferroptotic process decreases PMN-MDSC numbers, their immunosuppressive activity is enhanced due to the increased release of immunosuppressive molecules.^23^ Inhibition of ferroptosis by liproxstatin-1 could alleviate the immune suppression mediated by PMN-MDSCs and reduce tumor growth, especially when combined with immunotherapy.^23^ These studies indicate that ferroptosis in several immune cell types exerts tumor-supportive effects, raising the possibility for future investigation into selectively inhibiting ferroptosis in immune cells through cell-specific delivery to inhibit tumor growth effectively.

Ferroptosis inhibition counteracts the immunosuppressive activities of ferroptotic cancer cells to restrict tumor progression. For example, mutated KRAS protein derived from ferroptotic PDAC cells is packaged into exosomes, which are then engulfed by adjacent macrophages, promoting their subsequent polarization into an M2 tumor-promoting state.^321^ Administration of ferrostatin-1 can inhibit the tumor-promoting growth arising from peripheral blood mononuclear cell-derived macrophages (PBMCMs) in immunodeficient mice.^321^ However, the impact of ferroptosis inhibition on PDAC tumor progression has not been investigated in immunocompetent mice. It is crucial to consider that inhibiting ferroptosis may also suppress the tumoricidal effect of cancer cell ferroptotic death and affect the immunoregulatory role of immune cells within the TME. Therefore, the application of ferroptosis-based approaches in cancer treatment should be applied with caution. More studies are needed to fully understand the complex interplay between ferroptosis, tumor progression, and immune response, to make informed decisions regarding the application of ferroptosis modulation in cancer therapy.

Because of the link between the anti-tumor effects of traditional therapy and ferroptosis induction, as well as the potential tissue-damaging properties of ferroptosis, ferroptosis inhibition is considered an effective approach to suppress the side effects mediated by traditional therapy-induced ferroptosis. For instance, cisplatin-induced ferroptosis was implicated in chemotherapy-induced ovarian damage, and the administration of antioxidant NAC can alleviate cisplatin-induced toxicity in normal ovarian cells by inhibiting ferroptosis and oxidative stress.^475^ Cisplatin-induced acute kidney injury and doxorubicin-induced cardiomyopathy also could be inhibited by ferroptosis inhibitor ferrostatin-1.^476,477^ The FDA-approved iron chelator dexrazoxane protects against doxorubicin-induced cardiotoxicity by chelating mitochondrial iron.^114^ Moreover, ferroptosis is also implicated in radiation-induced intestinal injury, which can be alleviated by administering ferrostatin-1.^478^ Ferroptosis inhibition appears to be a potential target for mitigating treatment side effects, potentially enhancing treatment tolerance, and prolonging the life quality of patients. However, it also raises concerns regarding whether the administration of ferroptosis inhibitors to mitigate the ferroptosis-related side effects induced by traditional therapy might also inhibit its therapeutic effect. In addition, the occurrence of ferroptosis was observed in wasting tissues during advanced tumors cachexia. It is reported that tissue-infiltrating neutrophils-secreted LCN2 induces ferroptosis and wasting tissues in lung cancer cachexia. Liproxstatin-1 has been shown to mitigate tissue wasting in lung cancer cachexia, improve symptoms, and extend the survival of cachectic mice by inhibiting ferroptosis.^479^

Collectively, ferroptosis inhibition holds promise as a strategy for suppressing tumor growth, attenuating adverse effects of traditional therapies and improving cachexia. Ferroptosis inhibitors generally encompass four classes^120^: 1) Radical-trapping antioxidants including ferrostatin-1, liproxstatin-1 and vitamin E.^4,8,298^ 2) Iron chelators such as deferoxamine, cyclipirox, and deferiprone.^4,120^ 3) Inhibitors of ferroptosis-promoting enzymes, such as ACSL4 inhibitor (thiazolidinediones and triacsin C.^46,68^ 4) Inhibitors of protein degradation in the ferroptosis defense system, such as 5-(tetradecyloxy)-2-furoic acid (TOFA) and dopamine for preventing GPX4 protein degradation.^45,480^ Additionally, compounds, like CoQ_10_, N-acetylcysteine (NAC) and β-mercaptoethanol (2ME) that disrupt the pathways involved in ferroptosis excitation can also be potential inhibitors.^120^ Several clinical trials are pending to assess the effectiveness of ferroptosis inhibitors, such as deferoxamine (DFO) and deferasirox (DFX) in anti-tumor treatment (Table 3). However, further investigations are required to ascertain whether their anti-tumor potential is dependent on their ability to inhibit ferroptosis.

**Table 3:** Ferroptosis inhibitors used for cancer research

Drugs/Compounds	Target	Mechanism	Indication	NCT	Phase	Reference (PMID)
**RTAs**						
Ferrostatin-1	RTA	Inhibits lipid peroxidation	N/A	N/A	N/A	36996941
Liproxstatin-1	RTA	Inhibits lipid peroxidation	N/A	N/A	N/A	36385526 36973755 33311482
Vitamin E	RTA/ALOXs	Inhibits lipid peroxidation and may inhibit ALOXs	PDAC Prostate Cancer CRC NSCLC	NCT01446952 NCT00809458 NCT00905918 NCT01871454	I III I II	24439385 27159577
XJB-5-131	RTA	Nitroxide-based mitochondrial lipid peroxidation mitigators	N/A	N/A	N/A	27725964
JP4-039	RTA	Nitroxide-based mitochondrial lipid peroxidation mitigators	N/A	N/A	N/A	27725964
**Iron chelator**						
Deferoxamine	Iron	Reduces intracellular iron	TNBC HCC Solid tumors	NCT05300958 NCT03652467 NCT05184816	II I I	31519186
Deferasirox	Iron	Reduces intracellular iron	MDS	NCT00940602	II	32203980
2,2-bipyridyl	Iron	Reduces intracellular iron	N/A	N/A	N/A	22632970
Ciclopirox	Iron	Reduces intracellular iron	HCC	NCT00990587	I	19589922
**Enzyme inhibitors**						
Zileuton	5-LOX	Inhibits 5-LOX activity	NSCLC HNC	NCT00070486 NCT00056004	II II	18281656 22425913
Troglitazone	ACSL4	Inhibits ACSL4	Sarcoma	NCT00003058	II	27842070
Rosiglitazone	ACSL4	Inhibits ACSL4	Solid tumor Prostate cancer Sarcoma	NCT04114136 NCT00182052 NCT00004180	II III II	27842070
Pioglitazone	ACSL4	Inhibits ACSL4	Thyroid Cancers Breast cancer	NCT01655719 NCT05013255	II II	27842070
2-acetylphenothiazine	NOXs	Inhibits NOXs	N/A	N/A	N/A	28813679
GKT137831	NOXs	Inhibits NOXs	N/A	N/A	N/A	22632970
Linagliptin	DPP4	Inhibits NOX1-mediated lipid peroxidation	NSCLC	NCT03337698	II	28813679
Vildagliptin	DPP4	Inhibits NOX1-mediated lipid peroxidation	Thyroid Cancer	NCT02862470	N/A	28813679
Alogliptin	DPP4	Inhibits NOX1-mediated lipid peroxidation	N/A	N/A	N/A	28813679
Baicalein	ALOX	Inhibits 12/15-LOX	N/A	N/A	N/A	27037021
PD146176	ALOX	Inhibits 15-LOX-1	N/A	N/A	N/A	27842066
AA-861	ALOX	Inhibits 5-LOX	N/A	N/A	N/A	27506793
**Protein degradation inhibitors**						
Dopamine	Neurotransmitter	Increases the stability of GPX4	HNC	NCT02241083	IV	27793671
TOFA	ACC	Inhibits GPX4 degradation	N/A	N/A	N/A	27159577
**Others**						
β-mercaptoethanol	Reducing agent	Promotes cystine uptake through bypassing xCT	N/A	N/A	N/A	22632970
CoQ_10_/idebenone	Antioxidant	Inhibits lipid peroxidation	Breast cancer HCC	NCT00976131 NCT01964001	I III	31634900 27159577
NAC	GSH	GSH synthesis regulator	Lymphoma	NCT05081479	I	32203980
MUFAs	Fatty acids	Decreases oxidizable PUFAs	Malignancy	NCT00924937	N/A	30686757 31270077

*NCT* national clinical trial, *N/A* not applicable, *RTAs* radical-trapping antioxidants, *TOFA* 5-(tetradecyloxy)-2-furoic acid, *NAC* N-acetylcysteine, *MUFAs* monounsaturated fatty acids, *ALOXs* arachidonate lipoxygenases, *5-LOX* 5-lipoxygenase, *ACSL4* acyl-CoA synthetase long-chain family member 4, *NOXs* NADPH oxidase, *DPP4* dipeptidyl-peptidase-4, *ACC* Acetyl-CoA carboxylase, *GSH* glutathione, *xCT* system xc^-^, *PUFAs* polyunsaturated fatty acids, *PDAC* pancreatic ductal adenocarcinoma, *CRC* colorectal cancer, *NSCLC* non–small-cell lung cancer, *TNBC* triple-negative breast cancer, *HCC* hepatocellular carcinoma, *MDS* myelodysplastic syndromes, *HNC* head and neck cancer

## Markers of ferroptosis

A standardized set of identification hallmarks for ferroptosis enables us to determine its occurrence in physiological and pathological conditions, facilitating further investigation into its role in cancer. Currently, four classes of markers, including lipid peroxidation, mitochondria morphological alteration, gene expression change, and TFR1 re-localization, have been considered suitable for detecting and accurately distinguishing ferroptosis from other forms of RCD.^12^ First, the core of ferroptosis occurrence is the peroxidation of membrane-localized lipids and their lethal accumulation. There are five methods available to detect lipid peroxidation, including BODIPY 581/591 C11 fluorescent probes, lipidomics, thiobarbituric acid reactive substances (TBARS), malondialdehyde (MDA) and 4-HNE staining. Among those, flow cytometry following BODIPY 581/591 C11 staining is a sensitive and convenient method for detecting lipid peroxidation. Second, multiple organelles are involved in ferroptosis. Endoplasmic reticulum-related oxidative stress,^481–487^ mitochondria-induced cysteine starvation,^134,160^ lysosome dysfunction,^138,275,488–491^ peroxisomes-mediated ether lipids peroxidation,^52^ and Golgi stress-related lipid peroxidation all contribute to ferroptosis induction.^492,493^ Among these organelles, the morphological alterations in mitochondria, characterized by shrinkage, increased density, and decreased cristae, are considered the morphological features of ferroptosis and can be observed using transmission electron microscopy. Nevertheless, these mitochondria alterations are not specific, as they can be observed in oxidative stress and mitochondrial stress, and mitochondria are not indispensable for ferroptosis induction in some conditions.^12,481^ Third, specific gene expression changes, such as increased CHAC1, PTGS2, SLC7A11 and ACSL4 can be detected in cells undergoing ferroptosis. However, these changes may not be universally observed in all contexts of ferroptosis.^12,494^ Moreover, a recent study has identified hyperoxidized peroxiredoxin 3 (PRDX3) protein as a novel marker of ferroptosis in vitro and in vivo, specifically detectable in ferroptosis, rather than mitochondrial oxidative stress or other forms of RCD, such as apoptosis, necroptosis and cuproptosis.^495^ Fourth, the re-localization of TFR1, which imports extracellular ferric into cells by endocytosis, contributing to the liable iron pool required for ferroptosis, has been demonstrated as a marker of ferroptosis.^86^ The relocation of TFR1 from the region surrounding Golgi to the plasma membrane can be observed through staining with the 373-FMA antibody. This method provides a potential avenue for selectively staining ferroptotic cells in tissue sections.

To accurately determine the occurrence of ferroptosis, multiple markers are needed, which must be detected before cell demise. The selection of an appropriate time point is important in detecting ferroptosis markers. Among these markers, lipid peroxidation is essential, while other indicators may not be fully detected. Additionally, pharmacological rescue experiments using ferroptosis inhibitors are also crucial. In evaluating the efficacy of ferroptosis-based therapies, appropriate markers for detecting ferroptosis within tumor tissue are necessary. Although staining for 4-HNE, hyperoxidized PRDX3, MDA and TFR1 shows relatively promising prospects in their applicability for ferroptosis detection in tumor tissue sections, these markers could not be used in a living organism. Blood, urine, and feces are regularly checked in clinic practice, and whether or not using these samples to detect ferroptosis levels in patients is worth exploring, because ferroptotic cells could release some specific substances into their microenvironment. Deciphering the changes in iron, lipids, metabolites, and immune mediators may provide guidance for the development of these techniques.

## Conclusions and perspectives

Ferroptosis is a distinct form of cell death characterized by iron-dependent phospholipid peroxidation, which is strictly controlled at multiple levels. Pharmacologically targeting ferroptosis holds great promise as an anticancer strategy. However, to realize the prospects of ferroptosis drugs in clinical practice, several additional challenges remain to be overcome in future research.

Firstly, there is a lack of well-established animal models to evaluate cancer ferroptosis in vivo. Current models mainly rely on the use of FINs, such as IKE and cyst(e)inase,^43,53,192,465,496^ to treat xenograft tumors. However, the timing and frequency of administration vary in practice, and the side effects of long-term administration and potential drug resistance are not well understood. Additionally, generating CRISPR/Cas9-mediated GPX4 knockout cancer cells for xenograft models fails to evaluate the efficacy and safety effects of ferroptosis drugs. Therefore, standardized animal models are needed to improve our understanding of ferroptosis biology in cancer and ease the comparison of studies between research laboratories and clinicians.

Secondly, the complex biological effects of ferroptosis in cancer present another challenge. In some cases, ferroptosis induction initially promotes tumor formation but later leads to tumor cell demise and suppression.^21,319^ Tumor formation is a complex process involving metabolic disorders. For example, our team first put forward that melanoma is a metabolically driven and metabolically remodeled cancer.^497,498^ Given the coexistence of established tumor cells and cells transitioning into tumor cells,^23^ solely using ferroptosis inhibitors or inducers may not effectively control early-stage tumors. Therefore, it is crucial to determine the appropriate therapeutic time window for FINs.

Thirdly, the lack of effective and specific drugs that can safely induce ferroptosis in cancer cells poses an additional challenge. Although several compounds have been discovered to induce ferroptosis, their in vivo potential is limited due to poor bioavailability and insufficient targeting. Developing small molecules compatible with in vivo conditions and exploring targeted protein degradation technologies, such as proteolysis-targeting chimaeras (PROTACs)^499,500^ and lysosome-targeting chimaera,^499,501^ offer promising strategies. Moreover, reducing the toxicity of FINs remains a challenge in clinical oncology. Shifting the focus of developing ferroptosis-targeting drugs from completely abrogating master regulators, such as GPX4, to other controlling complexes with lower toxicity, and developing combination treatment strategies based on ferroptosis are all viable approaches to mitigate its toxicity.

Fourthly, ferroptosis induction may have negative impacts on anti-tumor immunity, posing a challenge in achieving complete tumor elimination. It is essential to promptly neutralize the factors that contribute to the immunosuppression induced by ferroptotic cancer cells. Moreover, FINs could potentially kill anti-tumor immune cells. Therefore, the development of cell-specific precision targeting strategies is crucial for maximizing the efficacy of ferroptosis-induced therapy.

Lastly, identifying the patient population that would benefit most from ferroptosis therapy is crucial for successful clinical trials. The sensitivity of different cancer types to ferroptosis varies based on tumor origin and genotype. Integrating genetic information from the cancer genome can aid in predicting tumor response to specific ferroptosis drugs.

In conclusion, we are on the verge of an exciting era in the realm of ferroptosis research. Overcoming the challenges above will pave the way for successful translation into clinical cancer treatment, enabling the development of personalized ferroptosis-related anticancer strategies. We anticipate that novel ferroptosis-based therapies, guided by standardized animal models and precise evaluation of therapeutic time windows, will be developed and implemented in the near future.
